# The role of redox-active iron, copper, manganese, and redox-inactive zinc in toxicity, oxidative stress, and human diseases

**DOI:** 10.17179/excli2025-8449

**Published:** 2025-07-25

**Authors:** Klaudia Jomova, Suliman Y Alomar, Richard Valko, Eugenie Nepovimova, Kamil Kuca, Marian Valko

**Affiliations:** 1 Department of Chemistry, Faculty of Natural Sciences, Constantine the Philosopher University in Nitra, Nitra, 949 74, Slovakia; 2 Doping Research Chair, Zoology Department, College of Science, King Saud University, Riyadh 11451, Saudi Arabia; 3 Department of Chemistry, Faculty of Sciences, University of Hradec Kralove, 50003, Hradec Kralove, Czech Republic; 4 Center of Advanced Innovation Technologies, VSB-Technical University of Ostrava, Ostrava-Poruba, 708 00, Czech Republic; 5 Biomedical Research Center, University Hospital Hradec Kralove, 5005, Hradec Kralove, Czech Republic; 6 Centre for Basic and Applied Research, Faculty of Informatics and Management, University of Hradec Kralove, Hradec Kralove, Czech Republic; 7 Faculty of Chemical and Food Technology, Slovak University of Technology, 812 37, Bratislava, Slovakia

**Keywords:** iron, copper, manganese, zinc, oxidative stress, human diseases

## Abstract

Given the key importance played by the redox-active metals iron (Fe), copper (Cu), and manganese (Mn) in vital cellular processes, such as DNA synthesis, oxidative phosphorylation, the detoxification of reactive oxygen species (ROS), and angiogenesis, it is not surprising that their dysregulation plays a causative role in many human diseases. The same applies to redox-inactive zinc (Zn), which is involved in numerous biological functions, and serves as a structural element, a catalyst, and a participant in both intracellular and intercellular signaling and in maintaining immune system function. An imbalance in redox active (Fe, Cu, Mn) or redox inactive (Zn) metal ions, whether in excess or deficiency, is harmful and may disrupt the structural, regulatory, and catalytic roles of various antioxidant enzymes (superoxide dismutases (SODs), catalase (CAT), glutathione peroxidases (GPxs)), proteins, receptors, transporters, alter sulfhydryl homeostasis, generate high levels of ROS (e.g., hydroxyl radicals by the Fenton reaction), initiate lipid peroxidation, cause DNA damage, and lead to cell death via mechanisms such as ferroptosis, cuproptosis, cellular senescence, or inflammation. Maintaining redox homeostasis is essential for regulating numerous cellular signaling pathways. Redox-sensitive signaling pathways, such as the nuclear factor kappa B (NF-κB), mitogen-activated protein kinase kinase (MAPK), and nuclear factor erythroid 2-related factor 2 (Nrf2) pathways, form an intricate network that governs cellular responses to redox metal-induced oxidative stress and inflammation. The Nrf2 pathway is primarily responsible for mediating antioxidant defenses, whereas the NF-κB and MAPK pathways play roles in proinflammatory and stress-related responses. Dysregulation of redox-active Fe, Cu, Mn, and redox-inactive Zn can alter epigenetic regulatory mechanisms such as DNA methylation, histone modification, and non-coding RNA expression. The dyshomeostasis of metal ions is closely related to the pathogenesis of lung, renal, and gastrointestinal diseases, neurodegenerative disorders (Alzheimer's disease, Parkinson's disease, and Huntington's disease), psychiatric conditions (schizophrenia), and various cancers. This review summarizes recent findings on the role of iron, copper, manganese, and zinc in maintaining physiological functions, redox homeostasis, and human diseases.

See also the graphical abstract(Fig. 1).

## Iron

Iron is the 26^th^ element in the periodic table. Its electronic configuration is [Ar]3d^6^4s^2^, which indicates that iron can participate in various redox reactions and catalyze the formation of reactive oxygen species (ROS) in biological systems. Iron typically exists in three oxidation states, ferrous Fe(II), ferric Fe(III), and, to a lesser extent, ferrate Fe(IV) (Valko et al., 2005[430]; Valko et al., 2016[429]). At physiological pH, Fe(III) tends to form insoluble oxyhydroxide polymers, whereas Fe(II) remains soluble. However, Fe(II) is not stable in aqueous environments and readily reacts with molecular oxygen, resulting in the formation of Fe(III) and superoxide radical anion (O_2_^• -^) according to the reaction of Fe^2+^ + O_2_ → Fe^3+^ + O_2_^• -^.

An average well-nourished adult human contains approximately 5 grams of iron. Iron is a vital irreplaceable metal necessary for the growth and survival of nearly all organisms. It is critical for oxygen transport and cellular energy generation in mitochondria, as well as for DNA synthesis/repair and many other biological reactions (Galy et al., 2024[150]). Iron deficiency is a prevalent condition that affects approximately 700 million individuals globally. The repercussions of iron deficiency can vary significantly, leading to issues ranging from anemia to cognitive impairments in developing children (You et al., 2025[469]).

Iron is a critical component of numerous redox enzymes involved in intermediary metabolism, as well as in the membrane-bound electron transport chains associated with respiratory processes and photosynthesis. Its electronic configuration, which maintains electron transfer reactions, is attributed to the extensive range of redox potentials for the Fe^2+^/Fe^3+^ couple, which spans from −300 to +700 mV. Owing to the low solubility of Fe^3+^, iron is predominantly sequestered by various proteins, including transferrin, lactoferrin, hemoglobin, haptoglobin, hemopexin, and lipocalin (Ongey and Banerjee, 2024[330]).

Acute iron toxicity is a rather uncommon condition and is primarily linked to hepatotoxic effects. An imbalance in iron levels, whether excess or deficient, can have detrimental effects on human health. Living organisms use different mechanisms to maintain iron status within safe limits. Increased iron levels have been correlated with various pathological conditions, such as liver and cardiovascular diseases, malignancies, neurodegenerative diseases, metabolic syndrome, hormonal imbalances, and immune disorders (Valko et al., 2005[430]).

### Iron metabolism 

Organisms bind iron to proteins, limiting its chemical reactivity and potential toxicity. It is thought that only trace amounts of iron remain free as nonchelated or loosely chelated (Valko, 2005[430]). Cellular iron levels must be maintained to meet their metabolic demands while not exceeding the cell's capacity to retain iron in a bound form. Iron metabolism is tightly regulated by proteins that cooperate through sophisticated and interconnected mechanisms at the cellular, systemic, and tissue levels. 

Approximately 65% of iron is bound to the hemoglobin of circulating erythrocytes; 10% is a constituent of myoglobin, cytochromes, and iron-containing enzymes; and 25% is bound to iron storage proteins, ferritin, and ferritin-derived hemosiderin (Valko et al., 2005[430]; Tandara and Salamunic, 2012[409]).

Ferritin (FT) is known as a high-capacity, low-affinity iron storage protein, as each molecule stores an average of 1000-1500 iron atoms (Theil, 2011[419]; Kell and Pretorius, 2014[242]), with a maximum capacity of approximately 4500. The heavy chain of ferritin (FTH) is responsible for ferroxidase activity, oxidizing ferrous Fe(II) to ferric Fe(III), whereas the light subunit (FTL) facilitates the deposition of iron in the form of inert inorganic ferrihydrite aggregates (Levi et al., 1994[268]). The function of ferritin extends beyond merely storing excess intracellular iron; it also facilitates the release of iron during periods of deficiency or increased demand, directing it to the labile iron pool (LIP) for reutilization (Galy et al., 2024[150]) through the process of proteolytic lysosomal degradation known as ferrinophagy (Mancias, 2014[286]). Ferritin autophagy involves the targeting of FT by the nuclear coactivator receptor 4 (NCOA4), which binds to FT and delivers it to the autophagosome. NCOA4 depends on iron levels and is degraded by the proteasome in iron-depleted cells (Mancias et al., 2014[286]). This process of regulating iron flux *via* FT contributes to maintaining the iron level in cells. In addition to intracellular localization, some cells secrete FT into the extracellular space in the form of extracellular vesicles, where it may function as a potential source of iron for neighboring cells (Truman-Rosentsvit et al., 2018[422]). A small amount of FT circulates in serum (sFT) (Tandara and Salamunic, 2012[409]) and is used as a clinical marker of iron deficiency (low sFT) and iron overload/inflammation (high sFT) (Kell and Pretorius, 2014[242]).

Another essential protein involved in iron homeostasis is transferrin (Tf), a high-affinity and low-capacity transport protein with a capacity of only two iron atoms per molecule. Tf circulates in plasma and is essential for delivering iron to all cells (Valko et al., 2005[430]).

Some cells have specialized functions and differ in how they handle iron. The leading site of iron utilization is the bone marrow, where erythroid precursors use iron in heme synthesis during erythropoiesis. Hepatocytes are the main site of iron storage (Valko et al., 2005[430]), and enterocytes and macrophages are involved in iron export (Camaschella et al., 2020[66]). Almost 90% of the daily iron requirement (20-25 mg/day) is recycled by macrophages upon phagocytosis of senescent and damaged erythrocytes, and only 1-2 mg of dietary iron is absorbed per day in the intestine, compensating for the corresponding amount of loss caused by menstruation bleeding and desquamation. Since humans lack regulated physiological mechanisms for iron excretion, body iron levels are primarily determined by the rate of dietary iron absorption in the duodenum and proximal jejunum, which is tightly controlled.

#### Intestinal iron absorption

Dietary iron occurs in two main forms, heme and nonheme. The nonheme iron Fe(III) is reduced by ferric reductases, e.g., the duodenal cytochrome B reductase (DcytB) (McKie et al., 2008[302]; Gunshin et al., 2005[174]), before it can be transported into the cytoplasm by the divalent metal iron transporter 1 (DMT1) expressed in the apical membrane of enterocytes (Gunshin et al., 1997[173]). Heme iron is absorbed more efficiently but constitutes only a small fraction of dietary iron. Heme iron can be transferred across the apical membrane *via* the heme carrier protein (HCP1) expressed in the small intestine (Shayeghi et al., 2005[386]), but the mechanism of heme iron uptake remains unclear and may involve receptor-mediated endocytosis (Gräsbeck et al., 1979[168]). The heme group in enterocytes is catabolized by heme oxygenase-1 (HO-1), which releases ferrous iron (Fe(II)).

**Figure 1:**
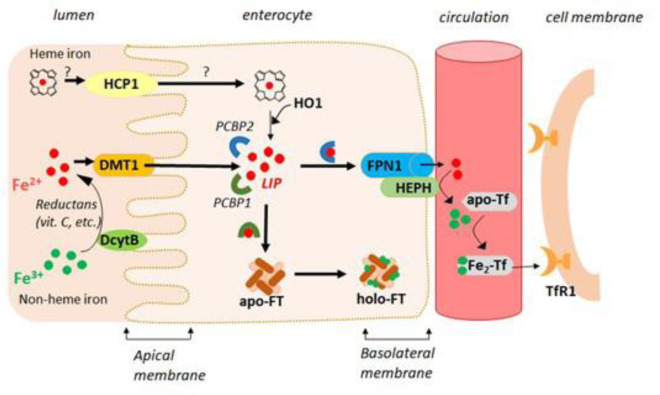
Graphical abstract. Schema of iron absorption through enterocytes. Heme iron can be transferred *via* the HCP1 transporter protein and degraded by HO1 in enterocytes, releasing ferrous iron (the putative uptake of heme *via* receptor-mediated endocytosis is not shown). DcytB reduces nonheme iron (Fe3+), and the formed Fe^2+^ is absorbed from the intestine by DMT1. The LIP is metabolically active iron, part of which is bound to chaperone proteins, PCBP1/2, delivering iron to FT (PCBP1) or FPN1 (PCBP2). HEPH oxidizes the iron released into the circulation through FPN1, and ferric iron (Fe^3+)^ is captured by Tf, forming a Fe_2_-Tf complex recognized by the ubiquitous cellular receptor TfR1. Abbreviations: HCP, heme carrier protein; HO, heme oxygenase; DcytB, duodenal cytochrome reductase; DMT1, divalent metal ion transporter; LIP, labile iron pool; PCB1/2, poly(RC)-binding proteins; FT, ferritin; FPN, ferroportin; Tf, transferrin; TfR1, transferrin receptor; HEPH, hephaestin.

Once inside intestinal epithelial cells, Fe(II) can be either transferred to the iron exporter ferroportin 1 (FPN1) at the basolateral side to release iron into the blood circulation (Donovan et al., 2000[123]; Donovan et al., 2005[124]) or sequestered in intracellular ferritin (Figure 1(Fig. 1)). Enterocytes retain iron when body stores are sufficient, and most iron stored in ferritin is lost via exfoliation of intestinal epithelial cells after the end of their life cycle (3-4 days) (Sharp and Srai, 2007[385]). Enterocytes may use some of the absorbed iron for their metabolic needs.

To prevent iron-mediated oxidative damage, the distribution of iron within enterocyte cells is mediated by the poly(RC)-binding protein 1 (PCBP1) and poly(RC)-binding protein 2 (PCBP2). PCBP1 binds iron with micromolar affinity and delivers it to cellular ferritin (Shi et al., 2008[389]) or nonhem iron enzymes (Nandal et al., 2011[321]) and is considered the major protein coordinating the “labile iron pool” (“free iron”) in the cytoplasm (Philpott et al., 2023[344]). PCBP2 facilitates iron intake and efflux through specific interactions with the DMT1 and FPN membrane proteins, respectively (Yanatori et al., 2016[463]). This chaperone protein can also interact with HO-1 to bind iron released from degraded heme (Yanatori, 2017[464]).

FPN1 is the only known iron exporter protein involved in the efflux of iron from cells (McKie et al., 2000[302]). Following iron export *via* FPN1 from enterocytes, Fe(II) is immediately oxidized to Fe(III) by the membrane-bound copper-dependent ferroxidase hephaestin (HEPH), a homolog of ceruloplasmin (Vulpe et al., 1999[436]), elsewhere in the body. Fe(III) is captured in the bloodstream by the iron-transport protein transferrin (Tf). Once iron has entered the circulation, there are no significant physiologic mechanisms for iron loss other than menstruation (Valko et al., 2005[430]).

The cells recognize circulating iron-loaded Tf via the ubiquitous transferrin receptor 1 (TfR1), which has a high affinity for ferric Tf (Wang et al., 2019[445]), and the “Fe_2_-Tf-TfR1” complex is internalized into cells *via* endocytosis (Mayle et al., 2012[299]). Tf is considered a regulator of iron homeostasis *by binding* to the TfR1 homolog TfR2, which has a lower binding affinity for iron-loaded Tf, and its expression is restricted mainly to hepatocytes and erythroid precursors (Silvestri et al., 2014[396]). At high plasma iron concentrations, Fe_2_-Tf binds to TfR2 and upregulates hepcidin (HEP) expression, possibly in a complex with hereditary hemochromatosis protein (HFE) (Kawabata et al., 2005[238]; Gao et al., 2009[154]). In erythroid cells, TfR2 and erythropoietin receptor (EpoR) are coexpressed during erythroid differentiation. TfR2 stabilizes the receptor on the cell surface (Forejtnikova et al., 2010[139]) and is regarded as a limiting factor for erythropoiesis, particularly in conditions of iron restriction (Nai et al., 2014[317]).

Regulation of intestinal iron absorption. Iron absorption in enterocytes is controlled by the coordination of regulatory mechanisms at the cellular and systemic levels. The liver-derived hormone hepcidin, a key regulator of systemic iron homeostasis (Nemeth et al., 2004[323]; Ganz and Nemeth, 2012[152]), is synthesized in response to high plasma iron concentrations and hepatic stores via the hemojuvelin and BMP/SMAD pathways (Silvestri et al., 2019[396]). HEP circulating in plasma binds to FPN, inducing the ubiquitination of FPN and the internalization and lysosomal degradation of the FPN-HEP complex. This process effectively reduces the plasma iron concentration by blocking the two primary routes of iron into the circulation-intestinal iron absorption and iron-recycling by macrophages-while intracellular labile Fe is retained (Nemeth et al., 2004[323]). The conditions under which hepcidin synthesis is repressed include iron deficiency, erythropoietic activity (Kautz et al., 2020[237]), and pregnancy (Sangkhae et al., 2020[367]) to increase iron availability. The other signal that promotes hepcidin synthesis is inflammation (Nicolas et al., 2002[326]). Systemic inflammation can trigger hepcidin synthesis by inducing the expression of proinflammatory cytokines, mainly IL-6, which activate *HAMP* (gene for HEP) expression (Wrighting and Andrews, 2006[455]), resulting in decreased iron absorption and sequestration of iron in macrophages, which explains the anemia observed in chronic disease (Weinstein et al., 2002[450]). Beyond the FNP‒HEP axis, further control of iron absorption occurs within enterocytes at every level of gene regulation (Figure 1(Fig. 1)).

In enterocytes, hypoxia-inducible transcription factor (HIF-2α), a mediator of cellular adaptation to hypoxia, has been shown to regulate iron absorption after changes in systemic iron levels *via* direct transcriptional activation of DMT1, DCYTB, and FPN (Shah et al., 2009[381]; Mastrogiannaki et al., 2013[296]; Taylor, 2011[415]). The regulation of this process is mediated by prolyl hydroxylase domain (PHD) enzymes, which require ferrous iron (along with molecular oxygen and 2-oxoglutarate) to hydroxylate HIF-2α. This modification renders HIF-2α susceptible to degradation. Thus, under hypoxia and low-iron conditions, reduced PHD activity stabilizes HIF-2α, enabling its translocation to the nucleus (Lee, 2018[263]). The promoter regions of DMT1, DCYTB, and FPN contain canonical hypoxia-responsive elements (HREs), and HRE-HIF2α interactions upregulate the genes of these iron transporter proteins to increase iron uptake.

Reducing iron levels in enterocytes enhances the binding of iron regulatory proteins (IRP1/2) to iron-responsive elements (IREs) present in untranslated regions (5´ or 3´ UTRs) of mRNAs encoding the key proteins of iron metabolism (Rouault, 2006[364]; Recalcati et al., 2017[358]). Under conditions of iron deprivation, when the LI level is reduced, IRPs are maintained in their active forms. The interaction of IRPs with the 3´UTRs of mRNAs for TfR and DMT1 protects the transcripts against endoribonucleases, increasing protein synthesis and iron uptake, whereas the binding of IRPs to the 5´UTRs of mRNAs for FPN and Ft represses their translation, which in turn inhibits iron export from the cell and iron storage, respectively. In iron-repleted cells, IRPs are inactive, followed by the synthesis of FPN and Ft and the degradation of mRNAs for TfR1 and DMT1. The posttranscriptional regulation of these proteins allows rapid adaptation to the Fe status and maintains the control of the pro-oxidant activity of increased LIP. 

Intestinal iron absorption involves crosstalk between IRP-hypoxia and the HEP-FPN axis (Camaschella, 2020[66]). The HEP-FPN axis is essential for intestinal HIF-2α activation and response under conditions of increased iron demand (Schwarz, 2019[376]). Under iron deficiency, the downregulation of HEP stabilizes FPN, promoting iron efflux. The decrease in iron in enterocytes stabilizes HIF-2α, leading to enhanced absorption.

### Iron-induced oxidative stress and toxicity

The cellular labile iron pool (LIP), also termed “free iron”, is a reservoir of chelatable and redox-active ferrous iron in the cytoplasm and functions as a pivotal junction in cellular iron metabolism (Kakhlon and Cabantchik, 2002[231]). This “form” of iron is loosely bound to carboxylates and phosphates of various low-molecular-weight ligands (e.g., glutathione, citrate, and amino acids) (Breuer et al., 2008[51]) and peptides (Krijt et al., 2018[256]), and some might exist as hydrated free iron. Because of the redox activity of the labile iron pool, it is thought to contribute to oxidative stress during iron overload (Piloni et al., 2016[345]). In mammalian cells, fluorescence analysis revealed that the labile iron concentration is less than 1 μM (Kakhlon and Cabantchik, 2002[231]). Labile redox-active iron can occur in plasma as a nontransferrin-bound iron (NTBI) that, under pathological conditions, induces oxidative damage (Breuer et al., 2000[50]). The cooperation of all protein molecules participating in iron metabolism is required to minimize the presence of free iron in the cytoplasm.

ROS-induced damage to important biomolecules is widely recognized as a significant mechanism contributing to the development of various chronic diseases (Valko et al., 2005[430]). The changes in cellular structure and function associated with iron overload appear to be intrinsically linked to the damage inflicted on cellular components and important biomolecules by ROS. Iron in the ferric state (Fe^3+^) has limited solubility at the neutral pH found in the intestine. Fe^3+^ tends to precipitate at physiological pH as polymeric ferric oxohydroxide complexes (soluble ferric ions are present in trace amounts of ^10-18^ M) (Kontoghiorghes and Kontoghiorghe, 2020[253]). Fe^3+^ is predominantly reduced to ferrous iron (Fe^2+^) by electron-transport ferric reductases, given that the absorption efficiency of ferric salts is approximately half that of ferrous salts.

The interconversion between the redox states of Fe^2+^/Fe^3+^ by biologically redox-active agents is fundamental to its absorption and biological properties and predetermines iron as a key component in the generation and metabolism of ROS in biological systems. Numerous in vitro studies have substantiated the generation of hydroxyl radicals (^•^OH), which can be elucidated through the following reactions (Valko et al., 2005[430]):


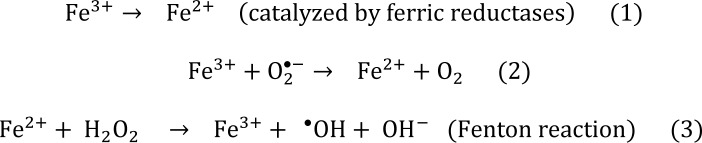


The overall reaction of steps (2) and (3) is termed the Haber‒Weiss reaction:


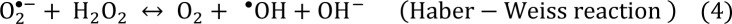


Furthermore, subsequent reactions may also occur


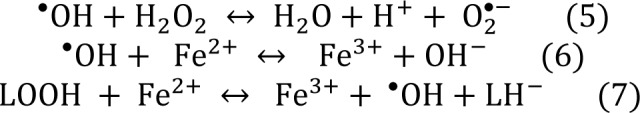


The decomposition of H_2_O_2_ in the presence of Fe(II), known as the Fenton reaction (3), produces ^•^OH, which can react with various biomolecules, including lipids, proteins, and DNA, at diffusion-limited rates (Lloyd et al., 1997[279]). Hydroxyl radicals are recognized as the most potent oxidizing agents in biological systems. They can trigger oxidative damage by abstracting hydrogen from amino-bearing carbons, resulting in carbon-centered protein radicals, and from unsaturated fatty acids, leading to lipid radicals (Chow, 1991[92]; Burkitt, 2001[59]). Additionally, these radicals can induce DNA strand breaks and base oxidation (Kawanishi et al., 1989[240]; Hayashi et al., 2000[187]; Buettner, 1993[55]).

To mitigate oxidative stress mediated by ^•^OH, shielding iron ions with a set of suitably selected ligands is necessary (Lawson et al., 2016[262]). The strong binding of low-molecular-weight chelators to iron through coordinating ligands such as oxygen, nitrogen, and sulfur inhibits the capacity of iron to facilitate redox reactions and act as a catalyst in the Fenton reaction. Given that iron has a maximum coordination number of six, it is often posited that hexadentate chelators can form more stable complexes by fully occupying the coordination sites of the iron atom (Lovejoy and Richardson, 2003[280]). As a result, a chelator that occupies all six coordination sites effectively neutralizes "free iron" in catalytic reactions. These chelators are referred to as "hexadentates," with desferrioxamine as a notable example. Numerous iron chelators exist that can inhibit the catalytic action of iron in various ROS-producing reactions. For example, desferrioxamine mesylate (DFO) significantly reduces the redox activity of Fe(III) and acts as a potent antioxidant because of its iron-binding properties (Valko et al., 2005[430]). In contrast, several iron-chelating ligands contribute to toxicity by promoting the generation of ROS through iron-catalyzed reactions (prooxidant behavior). For example, ligands such as nitrilotriacetate (NTA) and ATP are recognized for their ability to bind iron, leading to lipid oxidation and DNA damage. Additionally, the hexadentate ligand ethylenediaminetetraacetic acid (EDTA) has been shown to facilitate radical damage via Fenton chemistry.

The coordination of iron to biomolecules usually involves the involvement of the d orbitals of the metal. Molecular oxygen can interact with iron by utilizing the electrons in its antibonding π* orbitals, allowing for overlap with the d orbitals of the metal. Consequently, iron can simultaneously interact with both ligand atoms originating from biomolecules and molecular oxygen, functioning as a linkage between the biomolecule and oxygen and forming tetradentate complexes.

### Ferroptosis

Ferroptosis is a relatively newly discovered form of cell death that is dependent on iron and is characterized by increased oxidative stress and lipid peroxidation (Dixon et al., 2012[118]; Sawicki et al., 2023[369]). This process is intricately linked to both metabolic and ROS-mediated pathways. The accumulation of the labile iron pool resulting from increased iron uptake and heme degradation mediates the Fe-catalyzed decomposition of H_2_O_2_ via the Fenton reaction, thereby increasing lipid peroxidation (Fang et al., 2022[132]). The lipid peroxidation process results in the accumulation of lipid peroxides in the cellular membrane, increasing its permeability and, ultimately, cell rupture. This is accompanied by downregulation of the antioxidant enzyme glutathione peroxidase 4 (GPx4), which is tightly associated with myocardial infarction. In addition, ischemia/reperfusion is characterized by suppressed GPx4 and ferroptotic cell death.

### Iron-induced oxidative stress and human diseases

The capacity of humans to store iron is generally sufficient in healthy individuals. Iron in the circulation is predominantly bound to transferrin, resulting in only a minimal quantity of free catalytic iron under healthy conditions. However, as a result of oxidative stress, iron may be liberated from its protein complexes, potentially exacerbating oxidative damage to all important biomolecules. ROS-induced oxidative damage may play a significant role in the onset of various diseases, including cancer, neurodegenerative disorders, and cardiovascular diseases (Kawabata, 2022[239]).

#### Iron and cancer

Epidemiological trials have indicated a positive correlation between excessive iron consumption and the incidence of various cancer types. Hemochromatosis, a well-documented genetic disorder, results from iron accumulation, leading to liver cirrhosis or hepatocellular carcinoma due to iron-induced oxidative stress and cellular damage (Sung and Bae, 2014[404]). Several animal studies have demonstrated that the administration of iron via inhalation, intraperitoneal, or intramuscular routes can induce tumor formation in multiple locations. The primary mechanisms proposed for this phenomenon include oxidative damage to DNA, lipids, and proteins that initiates the carcinogenic process and the exacerbation of oxidative stress that disrupts antioxidant defense systems and associated signaling pathways, thereby facilitating abnormal cellular proliferation.

**Liver cancer.** As the liver is a primary reservoir for iron overload, this organ has been associated primarily with the etiology of hepatic carcinogenesis. Under physiological conditions, ferritin stores iron in a nontoxic form. However, iron dyshomeostasis leads to increased influx of iron, which in turn releases free iron from ferritin. Released iron can catalyze the formation of ROS, which can have detrimental effects on hepatocytes (Sung and Bae, 2014[404]).

The relationship between hepatic oxidative damage and the occurrence of nonalcoholic steatohepatitis (NASH) has been studied (Fujita et al., 2009[147]). Oxidative liver damage was assessed on the basis of the levels of 8-oxodG, a good marker of oxidative damage and one of the most common DNA lesions originating from the reaction between ROS and guanine. Compared with patients with simple steatosis, patients with NASH exhibit increased levels of oxidative damage. The level of 8-oxodG correlated with the formation of ^•^OH radicals, most likely via the Fenton reaction, and the extent of iron overload correlated with the clinical severity of hepatic steatosis in patients diagnosed with NASH. Iron chelation therapy was able to reduce the level of oxidative damage, accompanied by improved liver function.

As discussed above, iron homeostasis is critically regulated because of its significant toxic effects. Hereditary hemochromatosis (HH) is an autosomal recessive disorder characterized by hyperabsorption of dietary iron (Chen and Chloupková, 2009[78]). There are four distinct genetic variations associated with HH. Type 1 HH results from mutations in the HFE gene, which encodes a transmembrane human homeostatic iron regulator protein. Mutations in either the HAMP gene, which encodes the synthesis of hepcidin, or the HFE2 gene, which encodes hemojuvelin, a protein important in the regulation of iron homeostasis, characterize type 2 hereditary hemochromatosis, which is important in the development of cardiac and endocrine disorders. Type 3 hereditary hemochromatosis results from a mutation in the transferrin receptor 2 (TFR2) gene, which plays a crucial role in the regulation of hepcidin expression and iron transport. In contrast, Type 4 HH is attributed to a mutation in the ferroportin (FPN) gene, which encodes the duodenal iron-regulated transporter (Schimanski et al., 2005[375]).

**Breast cancer.** Iron-induced ROS formation in human breast cancer cells is supported by increased activity of heme oxygenase (HO-1) and consequently by increased expression of metalloproteinase-1 (MMP-1), suggesting a potential link between iron overload related to estrogen metabolism and cancer metastasis (Liou and Storz, 2010[275]). Excess iron is known to replace zinc in the N-terminal DNA-binding 'zinc finger' of the estrogen receptor, resulting in increased ROS formation. Analysis of breast tumor tissues confirmed the increased level of DNA damage, most likely attributed to the formation of 4-hydroxy estradiol (4-OHE) mediated by cytochrome P450, which in turn can trigger the formation of ROS such as H_2_O_2_ that are catalytically decomposed to ^•^OH radicals via the iron-Fenton reaction.

Elevated iron levels may correlate with increased breast cancer risk in postmenopausal women, most likely through the oxidative stress-induced activation of mitogen-activated protein kinase (MAPK) (Jian et al., 2011[213]).

Recent studies have reported that not only excess but also iron deficit is involved in breast cancer progression. Premenopausal women are often at risk of iron deficiency, and reduced levels of iron may play a role in angiogenesis (Huang, 2008[204]). This could increase the probability of breast cancer recurrence in premenopausal women compared with their postmenopausal counterparts.

**Colorectal cancer.** Experimental studies have provided compelling evidence regarding the significance of oxidative damage linked to metal toxicity and its potential carcinogenic effects in the colon, particularly concerning iron and copper (Buzard and Kasprzak, 2000[62]). Conversely, zinc is recognized for its antioxidant properties and may have a protective effect against chemically induced colonic preneoplastic changes in animal models.

The correlation between the intake of dietary iron and colorectal cancer incidence is ambiguous. A limited number of cohort studies have explored the positive correlations between the consumption of total iron, heme iron, red meat, processed meat, and heterocyclic amines and an increased risk of colorectal cancer (Cross et al., 2010[100]); however, multiple prospective cohort studies have reported no convincing associations between dietary iron intake and the risk of colorectal cancer (Zhang et al., 2011[478]).

**Figure 2:**
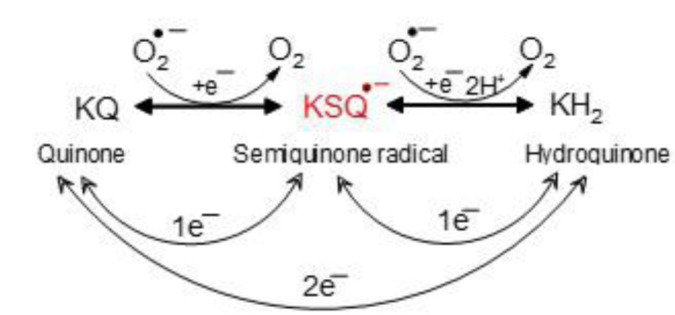
Interconversions of K vitamins among quinone, semiquinone radical, and hydroquinone forms.

We have proposed that secondary bile acids, specifically lithocholic and deoxycholic acid, along with K vitamins and iron(II) complexes, may interact to promote an oncogenic effect in the colon through the production of oxygen-free radicals (Valko et al., 2001[431]). Increased bile secretion due to a high-fat diet leads to increased synthesis of comutagenic and cocarcinogenic secondary bile acids by the microbiota present in the distal colon. Bile-tolerant strains of Bacteroides are capable of synthesizing and utilizing menaquinones, with vitamin K_1_ serving as a cofactor in the dehydrogenation process of bile acid substrates, resulting in the formation of reduced K vitamins (KH_2_) (Figure 2(Fig. 2)). These reduced vitamins may be transported into mature colonocytes in the form of mixed micelles, thereby initiating redox cycling reactions resulting in the formation of a semiquinone radical (KSQ^• -^). The EPR study confirmed the relatively high stability of semiquinone radicals in bile acid mixed micelles (Valko et al., 2001[431]).

**Renal cancer.** Recent research employing experimental animals has indicated that the administration of ferric nitrilotriacetate (Fe-NTA) results in deletions and single-nucleotide substitutions at G:C sites in renal cell carcinomas (RCCs). The kidney, an essential organ responsible for the excretion of urea, the reabsorption of important molecules, and the maintenance of ionic homeostasis, is known to significantly promote regeneration from residual tubular cells. This was documented by the heightened abnormal DNA replication and chromosomal missegregation of tubular cells exposed to chronic oxidative stress induced by the administration of ferric nitrilotriacetate (Akatsuka et al., 2012[5]).

#### Iron and neurodegenerative disorders

**Alzheimer's disease.** Alzheimer's disease (AD) is a neurodegenerative disorder characterized by progressive neuronal damage within the brain (Shippy et al., 2024[392]; Mezzaroba et al., 2019[306]; Chen et al., 2025[82]; Poprac et al., 2017[349]; Wang et al., 2024[446]). The hallmark features of AD pathology include the formation of soluble extracellular amyloid-β (Aβ) clusters that can circulate freely within the brain, eventually forming the amyloid plaques that are characteristic of Alzheimer's disease. Amyloid plaques are subsequently accompanied by the emergence of intracellular neurofibrillary tangles (NFTs) that consist of hyperphosphorylated tau protein (Holtzman et al., 2011[196]).

The triggering point of amyloid pathology is linked to the abnormal cleavage of amyloid precursor protein (APP) by β- and γ-secretases, leading to the production of Aβ peptides (Chen and Yan, 2010[80]), consisting of 39-43 amino acids (Selkoe, 1998[379]). The most abundant forms are Aβ40 and Aβ42 (Wang et al., 1996[444]; Butterfield et al., 2013[61]), of which Aβ42 tends to aggregate, forming the primary component of amyloid plaques in the brain (Miller et al., 1993[309]). The ratio of Aβ42 to Aβ40 is considered a critical factor in the pathogenesis of AD and is associated with increased neurotoxicity (Zhang et al., 2023[475]) and early-onset familial AD (Tanzi, 2012[412]).

The pathogenesis of Alzheimer's disease has been associated with a reduction in the neurotransmitter acetylcholine within the brain, attributed to the aberrant functioning of the enzyme acetylcholinesterase (AChE), which catalyzes the breakdown of acetylcholine into inactive metabolites, namely, choline and acetate. The primary treatment approach for individuals diagnosed with Alzheimer's disease involves the use of centrally acting acetylcholinesterase inhibitors aimed at mitigating the loss of acetylcholine in the brain (Hamulakova et al., 2016[181]).

Direct evidence indicating increased levels of oxidative stress in Alzheimer's disease encompasses several key observations: (i) dysregulated homeostasis of redox-active iron (Fe), copper (Cu) and nonredox zinc; (ii) redox-metal-catalyzed formation of ROS; (iii) elevated levels of lipid peroxidation products such as 4-hydroxynonenal (HNE), a byproduct of lipid peroxidation found in the ventricular fluid of AD patients; (iv) augmented levels of oxidized proteins and DNA; and (iv) impaired energy metabolism in the AD brain (Valko et al., 2005[430]). The neurotoxicity of amyloid-β is likely linked to its capacity to induce oxidative stress and its propensity to accumulate as deposits in the brain. Furthermore, Aβ has a strong affinity for metal ions such as zinc, copper, and iron, which may facilitate its aggregation. Numerous investigations involving Alzheimer's patients have demonstrated oxidative damage to Aβ, which correlates directly with the presence of free (unbound) copper and iron, both of which are redox-active metals. Copper strongly binds to the amyloid beta peptide through histidine (His13, His14, His6) and tyrosine (Tyr10) residues. Considerable advancements have been made in accurately measuring redox-active metals, specifically iron and copper, and the non-redox metal zinc within the brain tissue of individuals diagnosed with Alzheimer's disease. This progress has been facilitated by the utilization of three distinct physical methodologies: scanning transmission ion microscopy, Rutherford backscattering spectrometry, and particle-induced X-ray emission, in combination with a high-energy proton microprobe (Rajendran et al., 2009[355]).

These findings indicate a greater concentration of these metals within amyloid plaques in the brain tissue of Alzheimer's patients than in adjacent tissues. Specifically, the concentration of iron in amyloid plaques was found to be nearly twice that in surrounding tissue, whereas the levels of copper and zinc were approximately three times greater. These observations confirm the hypothesis that redox-active copper and iron play a catalytic role in generating oxidizing species, as manifested by oxidative stress markers in brain tissues. This finding supports a proposed mechanism whereby redox metals facilitate the production of free radicals, particularly hydroxyl radicals (HO^•^), primarily through the decomposition of hydrogen peroxide via the Fenton reaction (Zhao, 2019[480]).

Iron accumulation and oxidative stress have been identified as early phenomena in Alzheimer's disease (AD), suggesting that increased levels of redox-active iron may play crucial roles in promoting Aβ aggregation and oxidative damage associated with this condition (Everett et al., 2018[130]; Butterfield et al., 2013[61]).

Additionally, ferroptosis, a relatively newly identified form of iron-dependent programmed cell death (Dixon et al., 2012[118]; Masaldan et al., 2019[295]), is not only a result of lipid-based reactive oxygen species (ROS) accumulation but also a primary contributor to neuronal cell death in neurodegenerative disorders such as Alzheimer's disease.

Nevertheless, the exact origin of the redox-active iron found within the cores of amyloid plaques remains unclear. Various sources of iron, including ferritin, transferrin, and the labile iron pool, may contribute to the interaction between amyloid and iron in the context of Alzheimer's disease.

In a lipid environment, Fenton-like reactions involving Fe^2+^ species or other redox-active metals that are either free or coordinated with ligand L can react with lipid peroxides (ROOHs), leading to the formation of lipid alkoxy radicals (RO^•^) (Zhao, 2019[480])


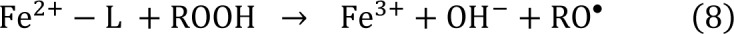


or more generally


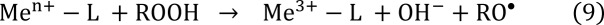


The experimental evaluation of DNA and RNA oxidation induced by hydroxyl radicals can be accomplished through the use of 8-hydroxy-2-deoxyguanosine (8OH-dG) and 8-hydroxyguanosine (8OH-G) as specific biomarkers (Bonda et al., 2014[41]). Research has indicated that elevated levels of 8OH-G are present in the cytoplasm of neuronal perikarya, which is associated with neurofibrillary tangles, a characteristic pathological feature of Alzheimer's disease.

Lysosomes occupy a central position in the homeostasis of iron and cover metabolic and cell death signals (Koskenkorva-Frank et al., 2013[254]). Most lysosomes contain relatively large amounts of macromolecules rich in redox-active iron and are unusually susceptible to cellular oxidative damage. The acidic and reducing environment in lysosomes results in the formation of ferrous (Fe^2+^) species, which in turn may catalyze the decomposition of H_2_O_2_ in the Fenton reaction and produce ^•^OH radicals. Unlike other organelles, lysosomes do not contain antioxidant enzymes such as superoxide dismutase, which converts O_2_^• -^ or catalase, which converts H_2_O_2_ (Cao et al., 2021[67]). This absence renders their membranes more susceptible to oxidative damage caused by ROS. ^•^OH radicals initiate the lipid peroxidation process of cellular membranes, which in turn may result in the release of iron into the cytosol and contribute to the formation of a labile iron pool (Kurz et al., 2011[257]). This cascade of reactions may ultimately cause severe damage to the cells responsible for apoptosis or necrosis, which is typical for neurodegenerative diseases. In addition, H_2_O_2_ increased lysosomal membrane permeability and induced apoptosis through the mitochondrial pathway.

**Parkinson's disease.** Parkinson's disease (PD) is a long-term, progressive neurodegenerative disorder that primarily affects movement (Zeng et al., 2024[473]). It is characterized by significant and selective degeneration of dopaminergic neurons in the nigrostriatal pathway. The clinical features of PD include various motor dysfunctions, including resting tremors, bradykinesia (a reduction in the speed of movement), postural instability, difficulties in gait, and muscular rigidity.

Three characteristic hallmarks of Parkinson's disease involve mutually interconnected pathologies: α-synuclein aggregation, dysregulation of autophagy, and mitochondrial dysfunction. α-Synuclein abnormally aggregates and accumulates in the form of Lewy bodies and Lewy neurites (Gómez-Benito M et al., 2020[165]). In addition, iron deposition in the substantia nigra (SN) is an important pathological alteration in individuals diagnosed with Parkinson's disease. In association with this, researchers have increasingly investigated therapeutic strategies aimed at addressing iron deposition in the context of PD (Zeng et al., 2024[473]).

**Figure 3:**
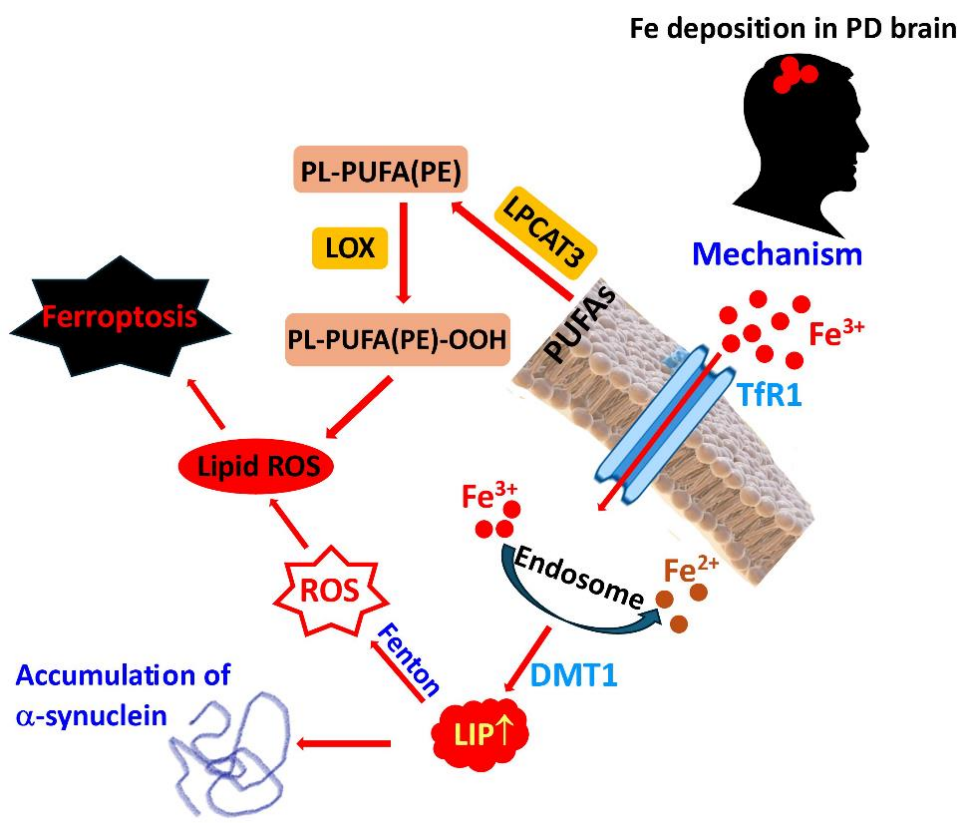
Roles of iron and ferroptosis in the pathology of brain cells in Parkinson's disease. Dietary iron enters cells in the ferric form (Fe^3+^) mainly through the transferrin/transferrin receptor 1 (TF/TfR1) transport system. Ferric iron is reduced by the metal reductase STEAP3, which has been identified in endosomes, where it collaborates with divalent metal transporter 1 (DMT1) to convert transferrin-bound Fe^3+^ into Fe^2+^, facilitating its transfer from the endosome to the cytosol. In the cytoplasm, Fe^2+^ initially forms a mixture of low-molecular-weight iron chelates. Once these complexes become saturated, any excess Fe^2+^ is stored in an unstable, so-called iron labile pool (LIP). The deposition of iron and α-synuclein aggregation are neuropathological hallmarks of Parkinson's disease. The Fe^2+^ from this labile iron pool can catalyze the decomposition of hydrogen peroxide in the Fenton reaction, producing reactive oxygen species (ROS), primarily hydroxyl radicals (^•^OH), which can lead to lipid peroxidation of membranes and ultimately result in ferroptosis. Lipid peroxidation serves as a critical signal that triggers oxidative damage to membranes during the process of ferroptosis. This phenomenon can be categorized into two types: nonenzymatic lipid peroxidation, also known as autoxidation, and enzymatic lipid peroxidation. In the case of autooxidation, reactive oxygen species (ROS) initiate the oxidation of polyunsaturated fatty acids (PUFAs), particularly arachidonic acid peroxide, resulting in the buildup of peroxides. Enzymatic lipid peroxidation is modulated by the activity of lipoxygenase (LOX), which catalyzes the formation of various lipid hydroperoxides from polyunsaturated fatty acids (PUFAs) (PL-PUFA(PE)-OOH). The enzyme lysophosphatidylcholine acyltransferase 3 (LPCAT3) is required for the biosynthesis and remodeling of polyunsaturated fatty acids. Phosphatidylethanolamine (PE) is a type of glycerophospholipid found within cellular membranes. In mitochondria, PEs constitute approximately 40% of the membrane composition, whereas in other organelles, they constitute approximately 20%. Abbreviations: LIP - labile iron pool; PL-PUFA - phospholipids containing a single polyunsaturated fatty acyl tail; LPCAT3 - lysophosphatidylcholine acyltransferase 3; PE - phosphatidylethanolamine; PL-PUFA(PE)-OOH - lipid hydroperoxide derived from PUFAs.

The precise mechanism of the abnormal accumulation of iron in the substantia nigra is not yet fully understood. Iron accumulation may not be a result of iron overload but rather is likely attributable to a disruption in iron homeostasis (Medeiros et al., 2016[304]). The correlation between iron deposition and the progression of Parkinson's disease remains ambiguous. Interestingly, in the initial clinical phases of disease progression, there is no marked iron accumulation in the ventral tegmental area, which is less affected than the substantia nigra pars compacta. However, as the disease progresses, iron deposition increases significantly (Zhang et al., 2023[474]; Jin et al., 2024[219]). The ferrous (Fe^2+^) ions present in this labile iron pool can facilitate the breakdown of hydrogen peroxide through the Fenton reaction, generating reactive oxygen species (ROS), predominantly hydroxyl radicals (^•^OH). This process can trigger complex lipid peroxidation reactions affecting cellular membranes and ultimately culminating in ferroptosis (Figure 3(Fig. 3)).

Iron serves a crucial function as an essential cofactor in neuronal metabolic activities (Ward et al., 2014[449]). An excess of iron, or iron overload, can catalyze the decomposition of hydrogen peroxide by the Fenton or Haber-Weiss reactions, resulting in the production of ^•^OH radicals and other ROS, which in turn mediate an increase in oxidative stress within tissues, cause cellular oxidative damage, and ultimately lead to cell death (Fischbacher et al., 2017[135]). Furthermore, abnormal iron accumulation may contribute to the degeneration of dopaminergic neurons in the substantia nigra through multiple pathways, including the induction of mitochondrial dysfunction and the facilitation of iron-dependent cell death (Zucca et al., 2017[484]; Wang et al., 2022[448]; Zeng et al., 2024[473]).

The central nervous system primarily acquires iron through a transmembrane carrier protein, transferrin receptor (TRF), and divalent metal transporter 1 (DMT1) (Jiang et al., 2017[215]). Disruptions in iron transport may contribute to irregularities in iron distribution. In Parkinson's disease, pathological α-synuclein proteins can influence iron homeostasis by modulating the ubiquitination process of DMT1 (Bi et al., 2020[35]). The neuroinflammation associated with α-synuclein accumulation leads to a decrease in key controllers of vertebrate iron metabolism, iron regulatory proteins (IRPs), and the iron-exporting protein ferroportin 1 (FPN1), which is essential for iron efflux from cells (Urrutia et al., 2013[428]). In the context of neuroinflammation, both TRF receptors (TRFRs) and DMT1 are upregulated, which enhances iron uptake by neuronal cells (McCarthy et al., 2018[300]).

In patients with Parkinson's disease, a marked reduction in the brain's levels of the iron-storage protein ferritin has been observed, accompanied by increased levels of total iron. Thus, ferritin may be instrumental in modulating iron metabolism in dopaminergic neurons (Kawabata et al., 2022[238]). Ferritin acts as a proinflammatory agent and sensitive biomarker of inflammation, demonstrating a significant association with the incidence of various diseases, including Parkinson's disease (Zhang et al., 2021[477]). The observed increase in the Fe^2+^/Fe^3+^ ratio in patients with Parkinson's disease has been associated with increased levels of ROS produced by the Fenton reaction (Wypijewska et al., 2010[457]). Structural changes resulting in an altered ratio of ferritin light chain protein to ferritin heavy chain protein, which is typical for PD, are associated with disturbed stability of iron storage, which in turn may increase the levels of unstable iron (Friedman et al., 2012[144]).

Iron accumulation activates microglia and astrocytes, which in turn results in the release of proinflammatory cytokines, contributing to increased oxidative stress levels (McCarthy et al., 2018[300]). An integral part of this process is neuroinflammation, deterioration of the functions of dopaminergic neurons, and progression of Parkinson's disease. In addition, accumulated iron can facilitate ferroptosis, contributing to disease progression (Dusek and Hofer, 2022[128]).

Taken together, the deposition of iron in the substantia nigra and other brain areas contributes to the progression of Parkinson's disease via several mechanisms, including iron-induced oxidative stress, iron-mediated cellular apoptosis, the aggravation of α-synuclein pathology, and the initiation of neuroinflammatory responses. Mitochondrial disruption of mitochondrial function is another key pathology contributing to disease progression.

Iron chelation therapy represents a potentially effective strategy for managing Parkinson's disease (Jomova and Valko, 2011[227]). The use of suitably designed iron chelators has been shown to decrease the accumulation of iron in the brains of PD patients, which may lead to enhancements in motor function (Devos et al., 2014[113]). A recent international, multicenter, phase II clinical trial revealed that the administration of iron chelators may exacerbate symptoms of PD in individuals with early-stage disease. This outcome highlights the need for further investigation into the role of iron deposition in the pathophysiology of PD.

#### Iron and cardiovascular disease

Iron's significant cardiovascular functions include the transport and storage of oxygen, the generation of energy, the formation of heme and iron-sulfur (Fe/S) clusters, and the catalysis of enzymatic reactions (Sawicki et al., 2023[369]).

Iron homeostasis is essential for maintaining cardiovascular health, as both iron deficiency and excess iron can adversely affect the cardiovascular system. The functionality of the cardiovascular system, particularly that of the heart, is intricately connected to mitochondrial performance, which is tightly linked with the availability of iron. Primary iron overload arises when the concentration of iron exceeds the intracellular storage capabilities of the body. The surplus iron may accumulate in multiple organs, resulting in dysfunction across various systems, with notable affinity for the heart, liver, and endocrine glands (Richardson et al., 2018[361]).

Some of the pathologies associated with iron overload affecting heart function include hemochromatosis and Friedreich's ataxia. Hemochromatosis is a hereditary disease characterized by impaired regulation of intestinal iron absorption. Approximately one-third of patients suffering from this condition exhibit cardiovascular disease, including arrhythmia, pulmonary hypertension, and heart failure (Udani et al., 2021[426]). Friedreich's ataxia most frequently manifests in early childhood and is characterized as an autosomal recessive disorder resulting from a mutation in the mitochondrial protein frataxin. A deficiency in frataxin disrupts the formation of iron‒sulfur (Fe‒S) clusters, adversely affecting numerous mitochondrial enzymes and leading to impaired mitochondrial respiration, increased oxidative stress, and iron accumulation (Petit et al., 2021[342]). The most prevalent cardiac complication associated with Friedreich's ataxia is hypertrophic cardiomyopathy, which affects nearly 80% of patients by early adulthood and is the leading cause of death in this age group (Monda et al., 2022[313]).

Mitochondrial iron levels are tightly controlled because iron is necessary for the formation of iron-sulfur (Fe/S) clusters and heme within the mitochondria (Ichikawa et al., 2012[208]). The ATP-binding cassette (ABC) transporter ABCB8 plays a critical role in the efflux of mitochondrial iron and is vital for maintaining normal cardiac function. Deletion of ABCB8 in the hearts of mice led to the accumulation of mitochondrial iron and the development of cardiomyopathy. In addition, mice lacking ABCB8 exhibited mitochondrial damage and increased levels of ROS-induced oxidative stress, which ultimately resulted in increased cell death. These findings underscore the importance of ABCB8 in ensuring normal cardiac function, regulating mitochondrial iron homeostasis and the physiological level of oxidative stress, and facilitating the maturation of cytosolic Fe/S proteins.

Iron homeostasis is disrupted in failing myocardial tissue (Sawicki et al., 2023[369]) While certain investigations of explanted human hearts from patients undergoing cardiac transplantation have revealed diminished mitochondrial iron levels, others have revealed elevated levels of mitochondrial iron and total cellular heme iron in failing human hearts (Sawicki et al., 2015[370]). An increase in mitochondrial iron in murine models following ischemia/reperfusion injury, as well as in human hearts affected by ischemic heart disease, has been reported (Chang et al., 2016[74]). Taken together, the regulation of cellular and mitochondrial iron stores is impaired in failing human hearts, with the specific nature of this impairment potentially varying according to the subtype of cardiomyopathy.

When the levels of circulating iron surpass the transport capacity of transferrin, nontransferrin-bound iron becomes prevalent in the bloodstream and contributes to the development of atherosclerosis and vascular impairment by facilitating the generation of ROS, inducing apoptosis, and attracting inflammatory cells (Vinchi, 2021[434]). Epidemiological research has further established a positive correlation between iron overload, iron-induced oxidative stress, and the severity of atherosclerosis (Wolff et al., 2004[453]).

Clinical data have confirmed a close correlation between pulmonary artery hypertension and iron deficiency; however, the mechanism has not yet been elucidated (Quatredeniers et al., 2021[353]). It has been proposed that the regulation of iron metabolism modulated by the synthesis of hepcidin may be involved (Ramakrishnan et al., 2018[356]).

Animal experiments revealed that the expression of ferroportin, a transmembrane protein that drives iron efflux outside of the cell, is accompanied by increased levels of endothelin-1, a potent endogenous vasoconstrictor. These two processes may be involved in the etiology of pulmonary artery hypertension (Lakhal-Littleton et al., 2019[259]).

In murine models of ischemia/reperfusion injury, increased mitochondrial iron levels trigger iron-mediated ROS overproduction (Verma et al., 2002[433]). This increase in ROS subsequently disrupts energy production and the inhibition of respiratory chain enzymes.

Other pathologies associated with chronic iron overload are heart block and atrial fibrillation (Siri-Angkul et al., 2018[398]). Iron affects L-type calcium channels, voltage-gated sodium channels, and ryanodine-sensitive calcium channels in cardiomyocytes. Iron can interfere with the binding of calcium to its intracellular sites, leading to a failure in the inactivation of calcium-dependent channels and calcium influx, which may in turn trigger arrhythmias (Gordan et al., 2018[167]). Iron overload can also decrease the levels and activity of sarcoplasmic/endoplasmic reticulum Ca^2+^ ATPase 2a (SERCA2a), another cause of arrhythmias and impaired cardiac relaxation.

### Iron-induced ROS and epigenetic alterations

Redox metals undergo redox cycling reactions accompanied by the production of ROS. ROS can upregulate epigenetic mechanisms, contributing to disease development. The epigenome encompasses all modifications to chromatin that regulate genome expression without a change in the DNA sequence. Epigenetic plasticity is facilitated by enzymes that create and remove covalent modifications to DNA and histones (Domann et al., 2021[122]). The principal molecular mechanisms that mediate epigenetic regulation of gene expression are DNA methylation, histone posttranscriptional modifications (methylation, acetylation, phosphorylation, ADP-ribosylation, ubiquitination, and SUMOylation), and the expression of noncoding RNAs (Cheng et al., 2012[88]). A growing body of evidence indicates that the writers and erasers of the epigenome are influenced by the redox and metabolic status of the cell and by environmental signals (Domann et al., 2021[122]). Redox metal-induced oxidative stress can result in DNA damage, including base modifications, deletions, and chromosomal rearrangements, which may hinder the ability of DNA methyltransferases to utilize DNA as a substrate, thereby affecting global or gene-specific methylation patterns (Tarale et al., 2016[413]).

Changes in DNA methylation predominantly occur at CpG sites located in the promoter regions of genes. The methyl group in 5-methylcytosine plays a crucial role in the binding of transcription factors and the transcriptional repressor methyl-CpG binding protein 2 (MeCP2) during sequence-specific DNA‒protein interactions (Nan et al., 1998[320]; Jones et al., 1998[228]).

The process of DNA methylation is influenced by the level of hypoxia-inducible factor 1-alpha (HIF-1α), whose activation through epigenetic mechanisms has been associated with the incidence of various cancers (Hwang et al., 2014[206]). For example, hypermethylation of the glutathione-S-transferase P1 (GSTP1) gene, which increases cellular vulnerability to DNA damage, has been observed in prostatic cancer (Nakayama et al., 2003[319]). Furthermore, methylation of the E-cadherin promoter has been identified in human colon and liver cancers. Methyl-CpG binding protein 2 (MeCP2) plays a crucial role in the epigenetic silencing of genes and mutations in the MeCP2 gene (Valko et al., 2006[432]). Promoters of the MeCP2 gene are significantly hypermethylated in neuronal tissues.

In addition to cancer, iron-mediated ROS formation is implicated in several other disease states that can be linked to epigenetic changes. Chronic iron exposure in colonocytes leads to significant hypomethylation of the epigenome (Horniblow et al., 2022[197]). The authors have observed abundant epigenetic changes within CG loci of nuclear factor erythroid 2-related factor 2 (NRF2) targets associated with increased iron-induced lipid peroxidation. The results obtained from both in vitro and murine intestinal models demonstrate a novel mechanism by which excess dietary iron alters the intestinal phenotype through epigenetic modification. Fe/ascorbate-mediated oxidative stress causes changes in the activities of antioxidant enzymes (SOD2, GPx) that are associated with epigenetic changes in promoter DNA methylation and the stimulation of inflammation markers (Yara et al., 2013[466]).

While iron deficiency blocks vital functions of iron-dependent epigenetic enzymes, excess iron deficiency can lead to the generation of damaging ROS such as O_2_^• -^ and ^•^OH, directly inhibiting the activity of histone demethylases by oxidizing Fe(II) to Fe(III) in their catalytic center (Polytarchou et al., 2008[347]). ROS can change the pattern of histone posttranslational modifications via direct ROS‒histone interactions (García-Giménez et al., 2021[155]).

Recent findings have revealed the significant role of intracellular labile iron in regulating the epigenome (Camarena et al., 2021[65]). The labile iron pool is an active portion of ferrous iron within the cell. It participates in redox reactions, such as the Fenton reactions, resulting in the formation of ROS (hydroxyl radicals). The activity of various epigenetic effectors that modulate the epigenome can be affected by the level of intracellular iron and subsequent ROS formation. Alterations in environmental conditions or cellular metabolism may lead to variations in the iron reservoir, thereby impacting the epigenome (Camarena et al., 2021[65]). Thus, labile iron emerges as a critical element influencing the regulation of the epigenome. The ten-eleven translocation (TET) enzymes are methylcytosine dioxygenases and are instrumental in DNA demethylation. TET enzymes, together with Jumonji-C (JmjC) domain-containing demethylases that regulate chromatin structure by altering the methylation process, represent two key categories of epigenetic regulators that involve iron (Farida et al., 2023[133]). Both families are classified within the superfamily of Fe(II) and 2-oxoglutarate-dependent dioxygenases, a substantial group of enzymes that necessitate Fe(II) for their optimal enzymatic function (Tsukada et al., 2006[424]; Li et al., 2015[269]). TET enzymes oxidize 5-methylcytosine into a transient intermediate, 5-hydroxymethylcytosine (5hmC). In addition to serving as a demethylation intermediate, 5hmC functions as a stable epigenetic marker that can also affect transcription (Zheng et al., 2024[481]). JmjC domain-containing demethylases are the primary enzymes responsible for demethylating histone lysine residues, thereby influencing chromatin accessibility and transcriptional regulation.

## Copper

Copper (Latin term Cuprum, Cu) is the 29th element in the periodic table, with an electronic configuration of 3d^10^4s^1^. The cuprous ion (Cu(I), Cu^+^) possesses a filled set of d-orbitals, containing ten *d*-electrons (3d^10^). In contrast, the cupric ion (Cu(II), Cu^2+^) has nine *d*-electrons in its d-orbital (3d^9^), one of which is unpaired. This unpaired electron renders bivalent copper paramagnetic, indicating that it is in the most stable oxidation state (Jomova and Valko, 2011[226]).

In addition to industrial applications, copper is vital for the physiological processes of numerous living organisms. The redox properties of copper make this metal very important in electron transfer reactions in biological molecules. Copper is an integral part or cofactor for various proteins, also known as oxidoreductases, that participate in essential biological processes, including superoxide dismutases (SODs), cytochrome c oxidase, L-ascorbate oxidase, ceruloplasmin, and other enzymes (Jomova et al., 2024[221]). These enzymes possess distinct types of copper centers that enable them to oscillate between cuprous and cupric redox states throughout the processes of catalysis and electron transfer. Copper enzymes are involved in various reactions, including oxidation, oxygenation, and the reduction of nitrogen oxides. They maintain redox homeostasis and neurological functions, photosynthesis, and many other important biological functions.

### Copper metabolism

The equilibrium between the intracellular and extracellular copper levels is maintained by cellular transport mechanisms that control uptake, export, and intracellular compartmentalization (Harris, 1991[184]). This equilibrium, which is crucial for balancing the essentiality and potential toxicity of copper, is established not only at the cellular level but also across tissues and organs (Bull and Cox, 1994[56]).

**Figure 4:**
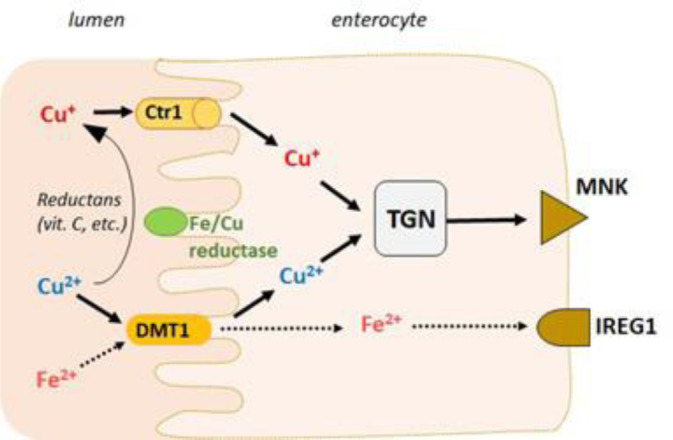
A simplified scheme of copper absorption by intestinal enterocytes. Divalent metal-ion transporter-1 (DMT1) and high-affinity copper transporter-1 (CTR1) play essential roles in the intestinal absorption of copper. Menkes protein (MNK; ATP7A) is a copper-transporting ATPase that has been found to be defective in the copper deficiency disorder Menkes disease. MNKs reside in a subdomain of the trans-Golgi network (TGN) and regulate copper homeostasis by translocating from a compartment localized within the trans-Golgi network to the plasma membrane in response to increased copper levels. The transport of ferrous ions (Fe2+) through the basolateral membrane is facilitated by a protein known as iron regulatory protein 1 (IREG 1).

Copper absorption in the human body is influenced by a variety of factors and dietary elements. Copper ions are carried in the bloodstream primarily through their association with proteins rather than being present in a free state. Approximately 75% of these ions are complexed with ceruloplasmin (CP) in a nonexchangeable form, whereas approximately 25% are associated with human serum albumin (HSA) in an exchangeable form. Additionally, approximately 0.2% of copper ions are bound to the amino acid histidine. Approximately 35-40% of the copper consumed is absorbed in the small intestine, with minimal absorption occurring in the stomach (Gaetke, 2003[148]). The primary mechanism for copper absorption involves an amino acid transport system, particularly the use of histidine, methionine, and, to a lesser extent, cysteine. Antioxidant 1 copper chaperone (Atox1) is a metallochaperone in humans that facilitates the transfer of copper ions from the primary plasma membrane copper transporter hCtr1 to the ATP7A/B proteins located in the Golgi apparatus (Figure 4(Fig. 4)) (Perkal et al., 2020[341]). This protein forms a dimeric interaction with the C-terminal domain of Ctr1, yet it delivers copper ions to ATP7A/B in a monomeric state. The dimeric structure of Atox1 contains a copper-binding site that includes cysteine residues Cys12 and Cys15, with lysine residue Lys60 also proposed to contribute to the copper-sequestering process. Liver hepatocytes play crucial roles in the absorption and storage of copper, in addition to regulating its excretion into the bile (Zhou and Gitschier, 1997[482]). The copper transporter also plays a role in the intracellular compartmentalization of this metal. Upon entering the cell, copper can be directed to (i) the metallothionein pool, (ii) the mitochondria for incorporation into cytochrome c oxidase, (iii) emerging Cu, Zn-superoxide dismutase (Cu, Zn-SOD), or (iv) the Wilson disease P-type ATPase located in the trans-Golgi network for subsequent integration into ceruloplasmin (Shim and Harris, 2003[390]). Ceruloplasmin accounts for approximately 95% of the copper present in serum.

The stringent regulation of copper homeostasis is crucial for preventing excessive accumulation of copper, making cases of acute and chronic copper toxicity relatively uncommon (Gaetke et al., 2014[149]). Copper that is absorbed beyond the metabolic needs of the body is typically eliminated through bile. The quantity of copper consumed through dietary sources and water is generally minimal, and the body effectively regulates excess copper levels by either reducing absorption or enhancing excretion under normal circumstances (Ratner and Rutchik, 2024[357]). Nevertheless, copper toxicity can occur due to excessive exposure resulting from industrial accidents, occupational risks, environmental pollution, and other routes of exposure. In addition, conditions such as adrenal gland insufficiency, genetic disorders affecting copper metabolism, and other contributing factors may cause copper-mediated toxicity. Recent research focused on the mechanisms by which copper imbalance occurs and its impact on metabolic processes has yielded valuable insights into the underlying pathophysiology, as well as potential therapeutic and preventive measures for health issues related to copper toxicity (Valko et al., 2005[430]).

### Cuproptosis

Recently, a newly discovered form of regulated cell death dependent on copper, termed cuproptosis, was identified (Tsvetkov et al., 2022[425]). The impact of cuproptosis on cell processes is very complex and may play a significant role in the development of various cancers.

Cuproptosis, an independent form of cell death, is highly correlated with mitochondrial respiration and the lipoic acid pathway (Tang et al., 2022[410]). The association between copper and cell death was reported as early as the 1980s; however, the exact mechanism was not known. Excessive intracellular copper can be transported to the mitochondria through ionophores, which are capable of reversibly binding copper ions, where it binds directly to the lipoylated component of the tricarboxylic acid cycle (TCA). The presence of copper ions can disrupt mitochondrial iron‒sulfur (Fe‒S) clusters, which are essential cofactors that mediate electron transfer within the mitochondrial respiratory chain (Wang et al., 2023[438]). This results in the excessive aggregation of lipoylated proteins, which are important components of the cuproptosis process. Inhibition of lipoylation may impede the progression of cuproptosis. Disrupted mitochondrial iron‒sulfur clusters are responsible for the induction of proteotoxic stress, ultimately resulting in cell death. Copper levels are significantly elevated in the serum and tumor tissues of patients with various malignant tumors compared with those in normal human serum and tissues. The phenomenon of cuproptosis underscores the critical role of cell death in the process of tumorigenesis, elucidating the mechanisms underlying malignant tumor development and advancement.

### Copper-induced toxicity and oxidative stress

Various transition metal ions, including iron (Fe) and copper (Cu), participate in redox cycling reactions, which facilitate the generation of ROS and RNS. Numerous investigations have linked the toxicity of copper to its ability to generate ROS, which can damage the most important biomolecules, including DNA, proteins, and lipid membranes (Halliwell and Gutteridge, 1984[180]). Interaction of cupric ions (Cu^2+^) with superoxide radical anion (O_2_^•-^) or reducing agents like ascorbic acid (AscH^-^) or glutathione (GSH) results in the formation of cuprous species Cu^+^


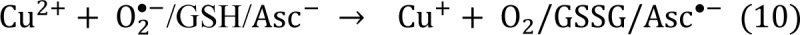


Cuprous ions can catalyze the production of hydroxyl radicals from hydrogen peroxide through the Fenton reaction (Haber-Weiss mechanism) (Bremner, 1993[49])


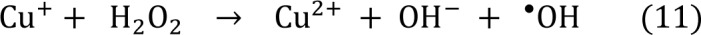


The hydroxyl radical exhibits a high degree of reactivity, enabling it to interact with nearly all biological molecules in close proximity through the hydrogen abstraction mechanism, which results in the formation of carbon-centered radicals, such as lipid radicals derived from unsaturated fatty acids. Copper in both cupric and cuprous states has shown greater efficacy than iron in promoting DNA breakage caused by the genotoxic metabolite of benzene, 1,2,4-benzenetriol. The primary mechanism of DNA damage is attributed to a site-specific Fenton reaction. Importantly, the upper limit of "free" copper pools within cells is significantly lower than one ion per cell, implying a considerable capacity for copper chelation within the cellular environment (Rae et al., 1999[354]).

Copper-induced toxicity is evidenced by the protective effects of metal chelators, such as bis-choline tetrathiomolybdate (TTM) and ethylenediaminetetraacetic acid (EDTA), which mitigate neuronal death in rats following intrahippocampal CuSO_4_ injections (Armstrong et al., 2001[12]). Long-term decreased levels of Cu,Zn-SOD (SOD1) are associated with impaired contraction of smooth muscle (determining the vascular tone of the blood vessel), most likely due to direct inactivation of nitric oxide production and an increase in lipid peroxidation (Lynch et al., 1997[282]). These findings indicate that dietary copper may influence endothelium-dependent arterial relaxation. Increased levels of Cu,Zn-SOD were accompanied by decreased levels of copper and ceruloplasmin, suggesting that ceruloplasmin may be a reservoir for the copper ions required for the synthesis of SOD1.

The generation of ROS due to copper exposure can lead to lipid peroxidation, which in turn elevates the concentration of the signaling molecule 4-hydroxy-2-nonenal (HNE) (Mattie et al., 2008[297]). HNE functions as a second messenger, potentially enhancing the phosphorylation and activation of the c-Jun N-terminal kinase/stress-activated protein kinase and p38 signaling pathways. The production of HNE is associated with increased activity of the AP-1 transcription factor and the upregulation of various genes, such as collagen type I, transforming growth factor beta 1, and gamma-glutamylcysteine synthetase. Additionally, HNE can promote c-Jun expression and activate protein kinase C (PKC) and the JNK/SAPK pathways.

The lipid peroxidation mechanism mediated by ROS is manifested by the oxidation of low-density lipoprotein (LDL). The resulting aldehyde products from the degradation of lipid hydroperoxides play crucial roles in altering the structure of the LDL apoprotein (Jomova et al., 2023[225]). Oxidized LDL can facilitate atherogenesis through the conversion of macrophages into foam cells, as well as the development of vasoconstrictive and prothrombotic characteristics. Although the in vivo studies are not fully convincing, in vitro experiments involving the incubation of LDL with copper ions have unequivocally demonstrated that copper induces LDL oxidation. Samples containing copper ions and displaying atherosclerotic lesions were identified by the generation of free radicals via copper ions. In addition to LDL, high-density lipoprotein (HDL) is also vulnerable to oxidation (Wang et al., 2023[438]). This is of key importance, as the oxidation of HDL is more susceptible to oxidative damage induced by copper than LDL is and markedly influences its cardioprotective functions. The mechanism behind this observation involves the activation of the so-called α-tocopherol-mediated peroxidation mechanism, wherein the oxidation of polyunsaturated fatty acids is initiated by the α-tocopheroxyl radical (α-toc-O^•^), which is produced during the reduction of Cu(II) to Cu(I) by α-tocopherol (α-toc-OH) (Jomova et al., 2023[225])


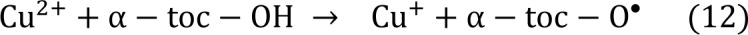


Furthermore, peroxynitrite, formed by the fast reaction between nitric oxide and superoxide


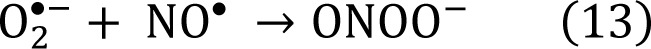


It is one of the most damaging species in biological systems. Peroxynitrite may facilitate the release of copper ions from copper-containing protein complexes such as ceruloplasmin (Swain et al., 1994[406]), which may in turn catalyze the formation of free radicals.

### Copper-induced oxidative stress and human disease

As discussed above, copper serves as a vital component within cellular structures, functioning as both an electron acceptor and a donor, thereby engaging in numerous biochemical reactions (Chen et al., 2020[79]). Copper metabolism requires precise regulation to maintain the homeostasis of this redox-active metal. An inherited or acquired imbalance in copper levels, whether it is deficient, excessive, or improperly distributed, can lead to or exacerbate various health conditions. The accumulation of copper ions mediates ROS formation, leading to excessive oxidative stress. Copper-induced oxidative stress is involved in the etiology of cancer, neurodegenerative disorders, cardiovascular disease, and other chronic disorders.

#### Cancer

As discussed above, copper in the human body exists predominantly in two oxidation states, with Cu^2+^ and Cu^+^ functioning as either electron acceptors or donors. This distinctive property allows copper to act as a crucial cofactor in redox reactions involving various essential enzymes, notably superoxide dismutase 1 (Cu,Zn-SOD, SOD1), which plays a role in the detoxification of ROS, and cytochrome c oxidase (COX), which is integral to the mitochondrial electron transport chain (ETC) (Ban et al., 2024[26]). Chronic copper overload or excessive copper accumulation can lead to oxidative damage and disrupt cellular processes. The oscillation of copper ions between oxidized and reduced forms produces OH^•^ via the Haber Weiss/Fenton reaction (Jomova et al., 2023[225]). Modified purine and pyrimidine bases represent a significant category of DNA damage induced by hydroxyl radicals, alongside other forms of damage, such as oligonucleotide strand breaks, DNA‒protein cross-links, and abasic sites in DNA (Dizdaroglu et al., 2002[119]). Thus, excess copper enhances ROS production and exacerbates oxidative stress, which can trigger DNA damage and mutations, culminating in genomic instability, the activation of oncogenes, and the deactivation of tumor suppressor genes. However, as cancer cells exhibit an increased demand for copper to support their vigorous metabolic activities, an overabundance of copper may lead to cell death due to cytotoxic effects stemming from increased mitochondrial-dependent energy metabolism and the accumulation of ROS.

Mechanistically, ROS can activate various signaling pathways that facilitate the initiation and progression of cancer, including but not limited to mitogen-activated protein (MAP) kinases (MAPKs) and the phosphatidylinositol 3-kinase (PI3K) pathway (Iqbal et al., 2024[210]).

Numerous studies have reported that cancer patients exhibit significantly increased levels of copper in both serum and tumor tissues (Zuo et al., 2006[485]). The application of highly specific atomic absorption spectroscopy and X-ray fluorescence revealed that while the concentration of copper is approximately 2 to 4 times higher than that of normal samples, the concentrations of physiologically related zinc, iron, and selenium are markedly reduced in cancer patients (Carpentieri et al., 1986[68]). Additionally, the ratios of Cu/Zn, Cu/Se, and Cu/Fe are generally greater in patients with malignancies than in healthy controls.

Copper-containing compartments have been shown to regulate the growth or regression of newly formed blood vessels (Gupte and Mumper, 2009[179]). Copper ions can promote the synthesis of fibronectin, a glycoprotein integral to the process of angiogenesis (Gullino, 1986[172]). Additionally, a decrease in copper levels has been associated with decreased expression of various crucial angiogenic cytokines and growth factors, including IL-1, IL-6, IL-8, b-FGF, TNF-α, and VEGF (Brem, 1999[48]). The evidence supporting the role of copper in tumor growth is further substantiated by the elevated copper levels found in the serum and tumors of cancer patients compared with those in healthy individuals. Increased copper levels in the serum or tissues of patients diagnosed with various cancers have been reported for colorectal, liver, and lung cancers (Vo et al., 2024[435]). Moreover, serum copper levels have been linked to tumor burden, progression, incidence, regression, and recurrence (Geraki et al., 2002[158]). In addition, elevated expression of ceruloplasmin in tumors has also been documented (Scanni et al., 1997[371]).

Current research interest examines the interplay between copper metabolism, oxidative stress, and the mechanisms by which copper exerts anticancer effects. These findings imply a significant correlation between copper, oxidative stress, and cancer (Xie et al., 2023[459]). Consequently, the strategic targeting of copper may offer a beneficial approach in the formulation of anticancer therapies that are linked to oxidative stress. A growing body of research highlighting the connection between cuproptosis and cancer progression has positioned this form of cell death as a focal point in cancer therapy investigations. Consequently, interventions utilizing copper to induce cell death present a promising avenue for the formulation of innovative anticancer strategies (Xu et al., 2023[460]).

Unlike many studies that have evaluated the effects of excess copper on oxidative stress, the effects of copper deficiency on organ functionality in cancer patients remain inadequately explored. The spleen serves as an essential organ in the immune system. Elevated levels of copper can result in increased levels of oxidative stress and toxicity within the spleen, causing a decline in its functional capacity. The effects of a copper-deficient diet on the spleen in murine models have been studied (Li et al., 2025[271]). Copper deficiency is manifested by histological impairment of the spleen; dysregulated expression of oxidative stress regulatory pathways such as Nrf2, Keap1, and HO-1; and altered expression of critical inflammatory enzymes (iNOS and COX2) and the transcription factor NF-κB, resulting in oxidative damage. Increased levels of oxidative stress are characterized by reduced levels of superoxide dismutase (SOD), glutathione (GSH), and total antioxidant capacity (T-AOC), alongside elevated levels of catalase (CAT) and mutagenic and carcinogenic malondialdehyde (MDA), a product of the lipid peroxidation process. A significant increase in the levels of inflammatory cytokines, including IL-1β, TNF-α, IL-6, and IL-8, was detected. Interestingly, copper supplementation reversed these alterations.

#### Copper and neurodegenerative disorders

Copper is an integral part of various proteins essential for neurological function. Disrupted copper homeostasis results in copper-induced oxidative stress. Its importance has been particularly noted in disorders such as Alzheimer's disease (AD), Parkinson's disease, and amyotrophic lateral sclerosis (ALS).

**Alzheimer's disease.** The main pathologies typical for Alzheimer's disease are discussed in the subchapter devoted to iron (see above). Briefly, Alzheimer's disease (AD) is the most common neurodegenerative disorder, affecting approximately 10% of individuals aged 65 and older and approximately 40% of those aged 80 and above (Zhang et al., 2011[478]).

The brain regions most affected by Alzheimer's disease are the cortex and hippocampus. These two regions accumulate extracellular senile plaques and intracellular neurofibrillary tangles (Zhang et al., 2011[478]). Senile plaques are composed of small peptides known as amyloid plaques (ANPs), which are formed from the Aβ precursor protein (APP). Neurofibrillary tangles (NFTs) are τ proteins predominantly found in neurons. The most significantly damaged region in AD is the hippocampus, the centrum of long-term memory, and the cortex, which is damaged by the presence of amyloid plaques implicated in cognitive functions (Leskovjan et al., 2009[267]).

Progress in the accurate quantification of redox-active copper and iron and non-redox zinc has been facilitated by the utilization of three distinct physical methodologies: scanning transmission ion microscopy, Rutherford backscattering spectrometry, and particle-induced X-ray emission, in combination with a high-energy proton microprobe (Rajendran et al., 2009[355]). These findings indicate a notable increase in the concentrations of these metals within the amyloid plaques of individuals diagnosed with Alzheimer's disease compared with those in adjacent healthy tissues. Specifically, the concentration of iron in amyloid plaques was found to be nearly twice that in surrounding tissue, while the levels of copper and zinc were observed to be approximately three times greater.

The association between the level of neurodegeneration and the potential for copper deposition has been studied in mouse models of AD (Bourassa et al., 2013[42]). The results demonstrated that copper accumulates in senile plaques in neurodegenerative models, such as the 5×FAD transgenic mouse model of Alzheimer's disease, which is characterized by early-onset amyloid pathology, and a mouse model frequently used in AD, CVN-AD,, which shows a premature reduction in neurovascular coupling. Conversely, no accumulated copper has been observed in the PSAPP transgenic mouse model, which shows only mild neurodegeneration (James et al., 2017[212]; Pal et al., 2014[334]). Data obtained from model systems support the observation that redox-active copper and iron cations and non-redox zinc cations are present at elevated levels within senile plaques in the brains of patients with Alzheimer's disease (AD) (Bagheri et al., 2018[19]).

Several different models regarding the role of copper toxicity in Alzheimer's disease (AD) have been proposed (Bush et al., 2003[60]). The most widely accepted model suggests that Aβ interacts with Cu^2+^ ions (Multhaup et al., 1996[315]). More recent theories (Cavaleri, 2015[71]) argue that Aβ may serve a protective function against the accumulation of harmful metals in the brain, suggesting that the loss of Aβ function could be a contributing factor to the pathogenesis of the disease (Keep, 2016[244]).

Copper and zinc may trigger self-assembly of Aβ into insoluble fibrils, as manifested by increased levels of these metals in Aβ plaques. Unlike Zn^2+^, redox-active Cu^2+^ is particularly important because of its ability to participate in redox cycling reactions, resulting in ROS formation (Abelein et al., 2022[2]). Thus, the neurotoxicity of Aβ is linked primarily to electron transfer reactions mediated by Cu^2+^ ions and subsequent ROS formation (Brewer, 2015[53]).

Paramagnetic NMR spectroscopy and molecular dynamic simulations revealed that monomeric Aβ interacts with Cu^2+^ at the N-terminal region of the protein, forming two predominant coordination modes for Cu^2+^.

Specifically, the interaction between Cu^2+^ ions and Aβ fibrils in the presence of biological reductants such as glutathione or ascorbate results in the formation of H_2_O_2_ (Parthasarathy et al., 2014[339]). Neurotoxic Aβ promotes the copper-mediated oxidation of ascorbate (AscH^-^), particularly in the central nervous system (200-400 μM). The findings of this study suggest that toxic Aβ peptides facilitate the oxidation of ascorbate through interactions with copper ions, leading to the production of hydroxyl radicals via the Fenton reaction (Valko et al., 2005[430]), the source of oxidative damage and neurotoxicity in the Alzheimer's disease brain. An increase in the copper-to-peptide ratio correlates with elevated levels of H_2_O_2_ and ^•^OH, resulting in a transition of aggregate morphology from fibrillar to amorphous structures (Mayes et al., 2014[298]).

Some findings suggest that the copper‒amyloid complex generates fewer ROS than free copper ions do. This is not surprising given that saturation of copper binding sites (copper has 6 binding sites) by donor atoms originating from Aβ reduces the catalytic activity of copper ions to generate ROS (Valko et al., 2005[430]; Nakamura et al., 2007[318]).

In vitro studies revealed that both oligomeric and fibrillar forms of β-amyloid can inhibit H_2_O_2_ production at elevated concentrations of Cu^2+^, with the fibrillar form producing less H_2_O_2_ than its oligomeric counterpart does (Fang et al., 2010[131]).

It has been proposed that the Cu^2+^ complex at the N-terminus is reduced by electrons originating from the C-terminal methionine (Met35) residues (Huang et al., 1999[205]), resulting in the formation of the sulfide radical of Met35 (MetS^• +^). This radical can further react with superoxide radicals to form Met-sulfoxide (MetO), which is found in the senile plaques of patients with Alzheimer's disease.

**Parkinson's disease.** The essential hallmarks of Parkinson's disease were briefly discussed in the previous chapter on iron. At this point, only the importance of redox-active Cu in the pathology of this disease is discussed.

The involvement of redox-active metals, particularly copper, in the etiology of Parkinson's disease has been substantiated by numerous epidemiological investigations (Bisaglia and Bubacco, 2020[37]). For example, prolonged occupational exposure to metals such as copper, iron, and other metals, either individually or in combination, even synergistically, has been linked to an increased likelihood of developing Parkinson's disease (Bjorklund et al., 2018[39]).

Copper and other redox metal-induced oxidative stresses are significant factors contributing to the degeneration of dopaminergic neurons in Parkinson's disease (Dias et al., 2013[115]). Alterations in the physiological regulation of redox potential within neurons disrupt various biological functions, ultimately resulting in cellular apoptosis.

The exact molecular mechanism by which copper dyshomeostasis may contribute to the development of Parkinson's disease is not fully understood. One of the possible mechanisms involves the ability of free (unbound) copper to interact with cysteine residues in proteins, leading to the potential suppression of their enzymatic functions. For example, rat striatal homogenates incubated with Cu^2+^ and other heavy metal cations exhibit significant reactivity toward thiols, resulting in a reduction in specific binding sites on D2 dopamine receptors (Scheuhammer and Cherian, 1985[374]). The administration of 3 mM copper was found to decrease the binding of D2 dopamine receptors with [3H]-spiperone by 40% to 60%.

Copper in the form of a labile copper ion pool as a result of metal dyshomeostasis has the capacity to oxidize dopamine, resulting in the formation of various potentially harmful species, including dopamine-quinones, superoxide radical anions (O_2_^• -^), hydrogen peroxide (H_2_O_2_), and hydroxyl radicals (^•^OH) (Monzani et al., 2019[314]). Copper-mediated oxidation of dopamine is predominantly achieved via copper coordination to various ligands, including amino acids and protein metal-binding sites. The catalytic activity of such copper species depends on the nature of the coordinating ligands, the stereochemistry around the copper ions, the number of binding sites, and other structural properties. Copper-mediated oxidation of dopamine is significantly influenced by the presence of the α-synuclein protein (Deas et al., 2016[109]). In vitro studies confirmed that α-synuclein-mediated ROS formation can be inhibited by the application of copper-chelating agents.

As outlined in the previous chapter dedicated to iron, the pathological buildup and clustering of α-synuclein, manifested as Lewy bodies and Lewy neurites, is a hallmark of Parkinson's disease. Specifically, the aggregation of α-synuclein is correlated with neuronal dysfunction and degeneration under these conditions. Initial in vitro studies have demonstrated that the presence of millimolar concentrations of various redox-active metal ions, including copper, facilitates the formation of partially folded amyloidogenic structures that exhibit a greater aggregation propensity (Binolfi et al., 2006[36]). While millimolar concentrations may not accurately reflect in vivo conditions, the pathophysiological importance of these findings is underscored by the localization of both α-synuclein and metal ions at synapses. The interaction between α-synuclein and metal ions may electrostatically stabilize a partially folded conformation of the protein (McDowall and Brown, 2016[301]). In addition to the C-terminal binding site, another copper-binding site with nanomolar affinity has been identified in the N-terminal region of the protein.

Parkinson's disease (PD) is characterized by the degeneration of dopaminergic and noradrenergic neurons, which are rich in neuromelanin and are located in the substantia nigra (SN) and locus coeruleus (LC), respectively (Weisman et al., 2024[452]). This neuronal loss leads to both motor and cognitive deficits. The most affected brain regions containing a high amount of neuromelanin and a high concentration of copper are the locus coeruleus and substantia nigra (Davies et al., 2013[106]). The increased levels of copper in these two brain regions are attributed to the unique metal-binding properties of neuromelanin (Bisaglia and Bubacco, 2020[37]).

**Amyotrophic lateral sclerosis**. Amyotrophic lateral sclerosis (ALS), which is frequently referred to as Lou Gehrig's disease or Charcot disease, represents the most prevalent lethal disorder affecting motor neurons (Cunha-Oliveira et al., 2020[105]). This neurodegenerative condition is characterized by the gradual degeneration of upper motor neurons located in the cerebral cortex, as well as lower motor neurons found in the brainstem and spinal cord. The resulting effects include muscle weakness, which ultimately progresses to muscle atrophy and paralysis.

While the mechanisms underlying amyotrophic lateral sclerosis remain elusive, considerable evidence suggests that an overproduction of ROS, coupled with a deficient antioxidant defense system, represents a significant pathological characteristic of the disease (Cunha-Oliveira et al., 2020[105]). ROS-induced oxidative stress has been associated with the degeneration of motor neurons and mitochondrial dysfunction, which are pivotal factors in the neurodegenerative processes associated with ALS.

Similar to other neurodegenerative disorders, abnormalities in redox metal homeostasis, which in turn trigger oxidative stress, may influence the onset and progression of ALS. In this context, redox copper has been the subject of extensive research and is recognized for its critical functions within the central nervous system. Copper plays a vital role in processes such as neurotransmission, angiogenesis, cellular respiration, and protection against oxidative stress. Copper dyshomeostasis can lead to functional deficits in the central nervous system, manifesting as neurodegeneration, a hallmark of amyotrophic lateral sclerosis (ALS) (Barros et al., 2018[29]).

Copper-induced oxidative stress is associated with alterations in gene expression, modifications in lipoprotein structure, changes in membrane receptor functionality, and subsequent impacts on lipid profiles. Whereas an overload of copper facilitates biochemical reactions that generate ROS, a deficiency of copper is manifested by diminished activity of copper-containing antioxidant enzymes, including superoxide dismutase (SOD), glutathione peroxidase (GSH-Px), and metallothionein (Uriu-Adams and Keen, 2005[427]). Thus, both pathological states, an excess or a deficiency of copper ions, are implicated in the increased level of oxidative stress, which is a pivotal factor in the progression of ALS.

Copper has the potential to affect lipid metabolism. The relationships among copper levels, lipid profiles, and functional status in individuals diagnosed with amyotrophic lateral sclerosis (ALS) have been the subject of study (Uriu-Adams and Keen, 2005[427]). Compared with those in healthy controls, the plasma copper levels in patients with ALS were significantly lower and were positively correlated with HDL cholesterol. Within the control group, plasma copper was positively correlated with serum ceruloplasmin, total cholesterol, low-density lipoprotein cholesterol (LDL-c), and HDL cholesterol. Notably, the serum ceruloplasmin level was positively correlated solely with the LDL-c level. In the case group, dietary copper intake, plasma copper, and total cholesterol were inversely related to the functional status of patients diagnosed with amyotrophic lateral sclerosis (ALS). This study revealed that patients with ALS have altered copper and lipid profiles, which may affect the functional capabilities of ALS patients.

**Copper and cardiovascular disease.** The precise regulation of physiological levels of copper in the bloodstream and organs is essential for numerous biological functions in mammals (Bajpai et al., 2023[24]). Disruptions in Cu levels can lead to adverse effects across various tissues and organs, resulting in a wide array of health disorders, including cardiovascular disorders (Medeiros, 2017[304]). Excess copper can be toxic, induce oxidative stress, and lead to atherosclerosis, heart failure, and life-threatening arrhythmias (Liu and Miao, 2022[278]).

As discussed above, the main reaction through which ^•^OH is formed is the Fenton reaction (reaction 3). The consequences of this reaction involve DNA damage, lipid peroxidation, and protein oxidation, which impede the activity of cellular proteases and restrict cell proliferation by interfering with the ubiquitin‒proteasome pathway (Seval and Beksak, 2018[380]). Furthermore, copper ions promote the aggregation of fatty acylated proteins and disrupt mitochondrial metabolic functions, thereby triggering cell cuproptosis through their interaction with fatty acylated proteins such as pyruvate, α-ketoglutarate, branched-chain-keto acid dehydrogenase, and the glycine cleavage system (Shen and Wang, 2023[388]).

Atherosclerosis is a significant cardiovascular pathology that encompasses conditions such as coronary heart disease, cerebrovascular disease, and peripheral arterial disease (Wang et al., 2023[438]). Although copper serves as a crucial cofactor for numerous enzymes, excessive copper accumulation can have detrimental effects, resulting in oxidative stress, toxicity, cellular injury, and potential cell death. Studies indicate that elevated serum copper levels are associated with a range of inflammatory vascular disorders, including atherosclerosis, which is positively correlated with an increased risk of coronary heart disease. The pathological mechanisms of atherosclerosis involve the following: (i) copper is a vital component of lysyl oxidase, promotes arterial endocardium expansion, and subsequently narrows the arterial lumen by accelerating the synthesis of extracellular matrix collagen (Ford, 2000[138]); (ii) copper-induced ROS formation and the oxidative modification of low-density lipoprotein cholesterol facilitate the development of atherosclerotic plaques (El-Hajjar et al., 2022[129]); (iii) the aberrant activity of ceruloplasmin, an acute phase reaction protein, exacerbates inflammatory responses (Neşelioğlu et al., 2022[325]); and (iv) Cu-induced oxidative stress is responsible for insulin resistance and the onset of diabetes, two pathologies closely associated with the progression of atherosclerosis.

The use of copper-chelating agents has been shown to inhibit the progression of atherosclerosis and inflammation (Magenta et al., 2014[285]). Specifically, the copper chelator tetrathiomolybdate (TTM) forms a complex with copper, effectively chelating it and preventing the progression of atherosclerosis, acute inflammation, and pulmonary fibrosis (Lawson et al., 2016[262]).

Myocardial ischemia/reperfusion injury is characterized by pathological events in which copper and oxidative stress play important roles (Zeller et al., 2022[472]). Copper has been shown to decrease the levels of iron‒sulfur protein clusters, triggering protein‒toxic stress responses that result in cellular apoptosis. Additionally, copper-induced oxidative stress enhances the oxidation of catecholamines and contributes to cardiotoxicity by promoting glutathione oxidation while inhibiting glutathione conjugation. Furthermore, copper disrupts lipid and fatty acid metabolism and ultimately causes damage to myocardial cells. Copper-containing ceruloplasmin is involved in the conversion of NO^•^ into NO_2_^-^ (nitrites), thereby exacerbating oxidative stress injuries in the myocardium.

Heart failure is a complex clinical condition resulting in compromised cardiac pumping ability and inadequate cardiac output, resulting in a failure to pump an adequate supply of blood to the body's organs and tissues (Girouard et al., 2025[162]). The key pathological mechanisms associated with heart failure involve copper dyshomeostasis, copper-mediated alterations in the cardiac energy supply by mitochondria, and copper-induced oxidative stress (Cooper, 2012[94]). A major copper-carrying protein in the blood activates hypoxic and ischemic signaling pathways, which in turn increase its expression and promote heart failure. Copper chelating agents have demonstrated the ability to restore mitochondrial function and enhance myocardial performance, thereby improving cardiac pump function (Barbariga et al., 2014[27]).

### Copper-induced ROS and epigenetic alterations

Copper can alter the epigenome *via* different epigenetic targets. The human hepatocyte cell line Hep3B showed a significant decrease in global histone acetylation at 100-200 μM copper through direct inhibition of histone acetylase activity (Kang et al., 2004[234]).

A study with an endoplasmic reticulum-associated protein (RTN1, Reticulon-1) focused on the inhibition of histone deacetylase (HDAC) enzymes has shown that the protein exhibits inhibitory activity only in the form of a copper complex (Nepravishta et al., 2009[324]). The Cu(II)-RTN1-C_CT_ complex induced dose-dependent inhibition of the histone deacetylase HDAC8 *via* oxidative damage and cleavage of the enzyme. These findings suggest that Cu-RTN1-C_CT_ could have therapeutic implications in cancer treatment since HDAC inhibitors exhibit powerful and selective anticancer properties in preclinical research (Shanmugam et al., 2022[384]).

PC12 cells are derived from rat pheochromocytoma and serve as a developed neuroendocrine cell model for investigating disorders affecting the nervous and endocrine systems. The impact of copper-induced ROS formation on the epigenetic modifications of PC12 cells was investigated (Sun et al., 2014[403]). These results suggest that copper activated the gene expression of DNA (cytosine-5)-methyltransferase 1 (DNMT1) and the NAD-dependent deacetylase sirtuin-1 (SIRT1). The activation was further enhanced by hydrogen peroxide. These observations can be explained by the redox properties of copper, which promote lipid peroxidation via the Fenton reaction via hydroxyl radicals. The application of the Fenton reaction system (copper and hydrogen peroxide) increased the expression levels of the DNMT1, DNMT3a, SIRT1, and SIRT2 genes in PC12 cells. Furthermore, resveratrol promoted the expression of SIRT1 through the deacetylation of p53, thereby reducing oxidative stress. The process of deacetylation serves to mitigate oxidative stress. Following treatment with diethyl phthalate (DEP), the expression of p53 was found to be elevated in PC12 cells. The elevated expression of SIRT1 noted in this study may play a role in providing protection against the oxidative stress induced by copper and hydrogen peroxide. Additionally, SIRT2 expression is also increased following exposure to hydrogen peroxide. The SIRT2 protein plays key roles in tumor-promoting and tumor-suppressing functions.

Wilson disease (WD) is a multifaceted disorder characterized by the accumulation of copper, primarily in the liver and brain. An interesting link between copper, genetic/epigenetic factors, and Wilson disease has been the subject of various studies (Medici and LaSalle, 2019[305]). The genetic foundation of this disorder is attributed to pathogenic mutations in the copper-transporting gene ATP7B, which leads to an impaired ability to excrete copper via the biliary system. Studies using animal models have demonstrated alterations in the regulation of methionine metabolism, which may influence DNA methylation processes. Studies involving mouse models of Wilson disease have revealed variations in the transcript levels of genes associated with DNA methylation in both fetal and adult liver tissues. The same conclusions have been reached in patients suffering from Wilson's disorder. However, the interplay between ATP7B genetic mutations and epigenetic modifications in relation to phenotypic outcomes requires further study.

Accumulated evidence suggests a link between epigenetic changes, such as DNA methylation, and tumorigenesis. The established role of copper in the modulation of tumorigenesis is mediated through both direct and indirect mechanisms, including its function as an angiogenic cofactor, its properties as a redox-active metal, and its involvement in the activation of several signaling pathways (Iftode et al., 2021[209]). The impact of copper (in the form of copper sulfate) on a human colon cancer cell line (HT-29) is manifested by altered expression of DNMT1 and DNMT3, thereby affecting DNA methylation. The data revealed an increase in the expression of these genes in response to copper exposure across a wide range of concentrations, thus confirming the hypermethylation effect of copper. A disrupted methylation regulatory cycle may, in turn, facilitate the proliferation of tumor cells.

Cupric ions have been reported to promote the epigenetic and metabolic remodeling of macrophages, driving them toward a proinflammatory phenotype (Flemming 2023[136]). Copper ions can influence the metabolism of monocyte-derived macrophages through the activation of H_2_O_2_, supporting the oxidation of NADH to NAD^+^. This process subsequently activates mitochondrial enzymes and induces metabolic reprogramming, which impacts the epigenetic regulation of proinflammatory genes by increasing the activity of histone demethylases and acetyltransferases.

## Manganese

Manganese is the 25^th^ element in the periodic table. Its electronic configuration is [Ar]3d^5^4s^2^, which indicates that manganese can participate in redox reactions and act as a catalyst in chemical reactions. Manganese is a highly versatile element that can exist in six distinct oxidation states. In its natural form, it is predominantly encountered in the reduced +2 state, which readily dissolves in water, or in the +4 state, which is known to form various types of insoluble oxides. Other common oxidation states of manganese are +3, +6, and +7, although all oxidation states from −3 to +7 have been observed. The manganese in the +7 oxidation state is represented by the purple permanganate anion MnO_4_^-^, a strong oxidation compound. Manganese is extensively utilized across a range of industrial applications. In manufacturing processes, Mn is integrated into the production of ceramics, steel, cosmetics, leather, fireworks, and glass (Chen et al., 2018[84]).

Manganese is a vital trace element found in all known forms of life. Numerous enzymes incorporate manganese as a key component. Manganese serves as a cofactor for various enzymes, such as manganese superoxide dismutase (Mn-SOD), arginase, and pyruvate carboxylase (Chen et al., 2018[84]). A notable example is the enzyme that facilitates the conversion of water molecules into oxygen during photosynthesis, which contains four manganese atoms.

The daily intake is approximately 2.5 milligrams, primarily sourced from foods such as nuts, bran, whole grain cereals, tea, and parsley. A manganese deficiency can lead to a reduction in bone density, increasing the susceptibility to fracture. Additionally, manganese plays a crucial role in the metabolism of vitamin B1 (Kippler and Oskarsson, 2024[249]).

### Manganese metabolism

Manganese is an essential mineral, yet it poses potential toxicity risks. Therefore, the body must meticulously manage manganese homeostasis. Although the precise mechanisms that regulate manganese homeostasis are not fully understood, systemic control is achieved primarily through the regulation of manganese absorption in the intestines and its excretion from the liver into the bile (Aschner and Aschner, 2005[13]).

In the bloodstream, manganese primarily exists in two oxidation states: Mn^2+^ and Mn^3+^. The Mn^2+^ form is found in dietary sources, is the most abundant form in circulation and is typically found complexed with various molecules, including albumin (which accounts for 84% of total Mn^2+^), hexahydrated ions (6%), bicarbonate (6%), citrate (2%), and transferrin (1%). Nearly all Mn^3+^ is associated with transferrin, contributing to its stability. Mn^3+^ is more cytotoxic than Mn2+ because of its increased oxidative reactivity (Chen et al., 2001[81]). This assertion was confirmed by a study involving rats administered either Mn^2+^ or Mn^3+^, which demonstrated increased signs of oxidative stress associated with Mn^3+^ (Ali et al., 1995[6]). Additionally, Mn^2+^ shows a limited affinity for sulfhydryl (-SH) groups and amines, and its stability constants remain consistent across various endogenous ligands, including glycine and cysteine (Aschner and Dorman, 2002[14]).

The reference ranges for manganese levels can differ between laboratories, with commonly reported values ranging from 0.9 to 2.9 μg/L (Burbee, 2009[57]). Serum manganese concentrations are typically lower than those found in blood, as approximately 85% of manganese is associated with hemoglobin within erythrocytes.

Manganese is taken up in the small intestine via an active transport mechanism and potentially through diffusion when dietary intake levels are elevated. Zinc transporter 8 (ZIP8) (encoded by SLC39A8) is a membrane transporter that has recently been recognized as a manganese transporter whose absence leads to significant manganese deficiency in humans, highlighting the essential role of ZIP8 in regulating manganese homeostasis within the body (Yu and Zhao, 2023[470]). The major transporter of manganese at the basolateral membrane is ZIP14 (encoded by the solute carrier 39 family member 14, SLC39A14 gene) (Scheiber et al., 2019[373]; Horning et al., 2015[198]). In addition, the entry of manganese into cells is mediated by transport proteins, such as the transferrin receptor and divalent metal transporter 1 (DMT1).

Iron significantly affects manganese homeostasis, as both elements utilize the transferrin (Tf) transporter and divalent metal transporter-1 (DMT-1) for binding and uptake. Trivalent manganese (Mn^3+^) occupies the same binding sites as trivalent ferric ions (Fe^3+^) on the large glycoprotein mucin, which plays a crucial role in stabilizing these ions and preventing their precipitation within the gastrointestinal lumen (Gunshin et al., 1997[173]). Additionally, both metals exhibit a strong affinity for the intracellular metal-binding protein mobilferrin. Manganese also shares iron lactoferrin receptors and ferroportin (Ye et al., 2017[468]). The absorption of manganese from dietary sources decreases with increasing iron meal content (Keen and Zidenberg-Cherr, 1996[241]). In addition, iron supplementation at a dosage of 60 milligrams per day over a four-month period is linked to lower blood manganese levels and reduced manganese superoxide dismutase (MnSOD) activity in leukocytes, suggesting a decline in manganese nutritional status (Davis and Greger, 1992[107]). Supplemental calcium (500 mg/day) and magnesium (200 mg/day) had a less pronounced effect on manganese absorption than did iron (Higdon et al., 2025[189]). Although manganese deficiency is relatively rare, severe manganese toxicity can be caused by overexposure via various routes.

When the transported manganese species are examined, the three most significant forms that penetrate the brain are Mn^2+^, Mn^2+^/^3+^ complexed with citrate, and the Mn^3+^-transferrin complex (Mn-Tf). Analysis of cerebrospinal fluid indicates that Mn-citrate is transported at the highest rates and represents the predominant Mn species that enters the brain (Crossgrove et al., 2003[101]; Chen et al., 2018[84]).

The release of manganese from cells is facilitated by several transport proteins, such as SLC30A10, which is a cell surface-localized manganese efflux transporter that maintains manganese at physiological levels and protects against manganese toxicity. In addition, manganese is removed from cells via the transmembrane (Madejczyk and Ballatori, 2012[284]). Additionally, subcellular organelles-including the nucleus, mitochondria, Golgi apparatus, lysosome, and endosome-employ different transporters for the intracellular movement of manganese; however, the precise regulatory mechanisms governing this process remain inadequately elucidated (Chen et al., 2018[84]).

### Manganese-induced oxidative stress and toxicity

The most frequent cause of manganese toxicity originates from exposure to manganese dioxide (MnO_2_) in mining operations or ore processing facilities. The neurological consequences of excessive manganese exposure in humans are predominantly linked to inhalation in workplace environments (Dorman, 2023[126]). High doses of manganese administered via several routes lead to manganese neurotoxicity, causing a condition known as “manganism”, which is characterized by behavioral alterations, tremors, facial spasms, irritability, hallucinations, and other symptoms, which may last from months to even years after exposure (Silva Avila et al., 2013[394]).

Manganese toxicity negatively affects mitochondrial functions by increasing ROS levels, hindering ATP synthesis, and modifying the structural integrity and permeability of membranes. This disruption can result in mitochondrial dysfunction, potentially leading to metabolic syndrome or other metabolic disorders. The development of metabolic diseases is directly associated with increased levels of oxidative stress. In addition, Mn toxicity involves autoxidation or turnover of intracellular neurotransmitter catecholamines and depletion of the cellular antioxidant network (Martinez-Finley et al., 2013[293]).

Manganese can participate in the Fenton reaction, resulting in the formation of ^•^OH; however, owing to its higher redox potential, it is less significant than iron or copper (Aguirre et al., 2012[4]). Manganese coordinated with certain organic ligands, such as bicarbonate, can catalyze the decomposition of H_2_O_2_ via a series of cyclic redox reactions (Archibald and Fridovich, 1982[10]).

**Figure 5:**
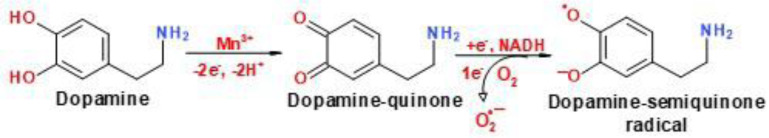
Oxidation of dopamine to dopamine-quinone by Mn^3+^ ions. Dopamine-quinone is transformed by NADH to dopamine-semiquinone radicals, which are unstable and transfer an electron to O2, producing O_2_^• -^.

Manganese preferentially accumulates in the substantia nigra (brain region affected by Parkinson's disease), which is rich in dopamine and particularly vulnerable to oxidative damage. ROS involved in the autoxidation of dopamine may cause damage to dopaminergic neurons (Archibald and Tyree, 1987[11]). As outlined above, trivalent manganese (Mn^3+^) is a considerably stronger oxidizing agent than its divalent form (Mn^2+^), and it has been demonstrated to effectively oxidize dopamine, resulting in elevated levels of harmful electron-deficient dopamine-quinone derivatives, which may covalently interact with nucleophiles, such as the thiol group in the amino acid cysteine (Cawte et al., 1989[72]). The oxidation of dopamine by Mn^3+^ in the absence of oxygen reduces its levels; this process can be inhibited by thiamine and ascorbic acid. Dopamine-quinone derivatives can be transformed via electron transfer reactions to semiquinone radicals by NADH, which is unstable and transfers an electron to molecular oxygen, producing a superoxide radical anion (O_2_^• -^) (Figure 5(Fig. 5)) (Cawte et al., 1989[72]).

The Mn^2+^ ion possesses a half-filled *d* shell, with 5 electrons and a similar spherical symmetry compared with those of Ca^2+^ and Mg^2+^ (Gunter et al., 1975[175]). The ionic radius of Mn^2+^ is greater than the ionic radius of Mg^2+^ and smaller than that of Ca^2+^. Thus, Mn^2+^ interferes with the role of Ca2+ in the activation of ATP via oxidative phosphorylation.

Cells and isolated mitochondria treated with Mn^2+^ inhibited ATP synthesis via oxidative phosphorylation, ultimately resulting in O_2_^•−^ production (Roth et al., 2002[362]). This and other studies confirmed that manganese induces ROS formation and mitochondrial opening of the permeability pore, contributing to a specific apoptotic pathway and resulting in cell death (Gunter et al., 2012[176]).

Mn toxicity is closely related to its role in promoting the production of ROS within several cellular compartments, predominantly through the mitochondrial electron transport chain (ETC), NADPH oxidases, and the enzymatic activation of cytochrome P450, xanthine oxidase, and cyclooxygenases 1 and 2. The primary formed radical is the superoxide radical anion (O_2_^• -^) in the mitochondrial matrix, which is dismutated by Mn-SOD (SOD2) (Lambert and Brand, 2009[260]). In the presence of manganese, hydrogen peroxide formed from O_2_^•−^ by the catalytic action of catalase-can be converted into the highly reactive and damaging OH^•^ (Goldstein et al., 1993[164]). ROS formation is evaluated via the use of 2′,7′-dichlorofluoroscein, a fluorescent probe, to detect ROS in biological systems following the addition of manganese (Chen et al., 2010[85]).

It has been established that intramitochondrial Ca^2+^ triggers a cascade of processes within the mitochondrion that increase ATP synthesis through oxidative phosphorylation, the activation of three dehydrogenases in the tricarboxylic acid (TCA) cycle, which facilitates increased generation of NADH and consequently the transfer of electrons to an acceptor such as O_2_ (Gunter et al., 2004[178]). The increased production of mitochondrial superoxide (O_2_^•−^ ) is accompanied by an increase in the membrane potential (Δψ) or the difference in hydrogen ion concentration across a membrane (electrochemical proton gradient, Δμ_H+_) (Hoffman and Brookes, 2009[193]; Hoffman et al., 2007[194]). The process of ATP generation through oxidative phosphorylation reduces the values of these gradients, which in turn suppresses ROS production (Brand, 2010[45]). 

Mn-induced toxicity is associated with Ca^2+^ mitochondrial transport. The mitochondrial calcium uniporter is a transmembrane protein that facilitates not only the transport of Ca^2+^ ions but also that of Mn^2+^ ions from the cytosol of a cell into the mitochondria (Gunter and Puskin, 1972[177]). In addition, Mn can disrupt energy metabolism and inhibit oxidative phosphorylation (Roth et al., 2002[362]). Mn^2+^ is not expelled from the mitochondria of neuronal cells by the predominant mitochondrial Ca^2+^ efflux mechanism (the Na^+^/Ca^2+^ exchanger) but rather by the Na^+^-independent Ca^2+^-efflux mechanism (Gavin et al., 1990[157]). The subsequent accumulation of Mn^2+^ in brain mitochondria represents the first site of manganese toxicity and is associated with a relatively long half-life of manganese in the brain.

Excess manganese primarily accumulates within the substantia nigra, striatum, and pallidum. The laboratory-generated mice were treated for two weeks with various concentrations of MnCl_2_.to assess cellular conditions, GSH levels, and ROS, as well as to evaluate the mRNA and protein expression of excitatory amino acid carrier 1 (EAAC1) and sodium-independent cystine-glutamate antiporter (xCT) (Yang et al., 2018[465]). The results indicated that manganese reduced the protein and mRNA expression levels of EAAC1 and xCT. In addition, increased ROS levels and the levels of oxidative stress markers, such as malondialdehyde (MDA), carbonyl, and 8-OHdG, increased in a dose-dependent manner. The accumulation of manganese in the striatum (a major subcortical structure of the forebrain), resulted in significant pathological and ultrastructural alterations.

The role of manganese in the modulation of nitrosative stress has been investigated (Bae et al., 2006[18]). Manganese (500 µM) upregulated iNOS expression and increased the phosphorylation of the ERK/JNK/MAPK and AKT pathways. In addition to the p38 MAPK-specific inhibitor SB203580, inhibitors of ERK, JNK, and PI-3 significantly reduced the protein and mRNA expression of iNOS. These findings suggest that manganese upregulates the expression of iNOS at the transcriptional level in murine microglial (BV2) and murine macrophage (RAW264.7) cells.

The mechanisms of Mn-induced neuronal apoptosis are multifaceted and involve the generation of ROS, protein aggregation associated with autophagy, and endoplasmic reticulum stress, collectively exacerbating nerve cell damage (Liu and Ju, 2023[276]). These cellular processes can have dual roles in either protecting or injuring cells under pathological conditions, potentially by interacting with one another. While endoplasmic reticulum stress may be associated with the compensatory activation of autophagy to increase cell survival, the precise relationship between these two phenomena in the context of Mn-induced neurotoxicity remains unclear.

A recent study reported that cells treated with varying concentrations of manganese chloride (MnCl_2_) resulted in severe cellular damage and apoptosis, significantly increasing the expression of proteins associated with the mTOR pathway (Cen et al., 2024[73]). Notably, the phosphorylation levels of the downstream targets 4E-BP1 and S6K1 and the highly related but functionally distinct kinases Akt and SGK1 were substantially reduced following mTOR inhibition, which was in agreement with a decrease in cell apoptosis. Moreover, the mTOR-specific inhibitor rapamycin was found to enhance learning and memory capabilities in vivo. Thus, mTOR inhibition may represent a potential therapeutic strategy for mitigating manganese-induced neurodegeneration.

Leucine-rich repeat kinase 2 (LRRK2) is a large multidomain protein that plays a significant role in inflammation and neurotoxicity in autosomal dominant and sporadic forms of Parkinson's disease (Bentley-DeSousa et al., 2025[34]). LRRK2 is highly expressed in microglia and macrophages and has been shown to interfere with manganese. Mn-induced toxicity was studied in human microglia (HMC3), active wild-type LRRK2 protein, and LRRK2-knockout (KO) RAW264.7 macrophages (Kim et al., 2019[245]). Mn activated LRRK2 kinase via phosphorylation of the serine 1292 residue, and two exceptionally efficient inhibitors (GSK2578215A and MLi-2) of LRRK2 were able to inhibit its manganese-induced activation manifested by suppressed apoptosis in microglia and macrophages and decreased Bax, a proapoptotic member of the Bcl-2 family. In addition, deletion of LRRK2 reduces ROS formation and the proinflammatory cytokine TNF-α (Jomova et al., 2025[222]). Manganese-induced toxicity is associated with LRRK2 kinase activity through downstream activation of MAPK signaling. These results indicate that LRRK2 may serve as a target for the development of therapies against Mn toxicity in neurodegenerative disorders.

Collectively, manganese induces oxidative stress and toxicity in two ways: by upregulating the prooxidant mechanism and by downregulating antioxidant mechanisms (Martins et al., 2025[294]). The promotion of prooxidant mechanisms includes (i) electron leakage from the transport chain, resulting in the formation of O_2_^•−^ ; (ii) the oxidation of dopamine; (iii) the limited efficiency of the Fenton reaction; and (iv) the upregulation of iNOS, resulting in the formation of NO^•^. Conversely, the downregulation of the antioxidant system following manganese exposure results in the inhibited activity of (i) antioxidant enzymes such as superoxide dismutase (SOD), catalase, and glutathione peroxidase (GPx) and (ii) protein inhibition involved in the synthesis of GSH.

### Manganese and human diseases

**Neurodegenerative disorders**. Manganism (locura manganica) is attributed to the selective accumulation of Mn in brain regions that are abundant in dopaminergic neurons, such as the substantia nigra (Aschner et al., 2005[15]). Manganese can oxidize catecholamines, most importantly the neurotransmitter dopamine. Fluctuations in the levels of striatal dopamine likely account for the biphasic syndrome, which is characterized by two distinct phases or components. The progression of manganism results in a decrease in catecholamine levels, likely due to the degeneration of nigrostriatal dopaminergic (DAergic) neurons, leading to symptoms similar to those of Parkinson's disease (Aschner et al., 2007[16]).

Numerous studies have shown that both toxic and subtoxic concentrations of manganese can influence key cellular signaling pathways that govern cell survival, differentiation, and apoptosis (Miah et al., 2020[307]). These pathways and neuroinflammation triggered by manganese play significant roles in the pathophysiology of neurodegenerative disorders, such as Alzheimer's, Parkinson's, and Huntington's diseases, as well as in other brain-related pathological conditions.

The neurotoxic effect of manganese is attributed predominantly to the release of proinflammatory cytokines such as interleukin 1-beta (IL-1β) and tumor necrosis factor (TNF-α) by microglia and astrocytes (Guilarte et al., 2010[171]). Astrocytes treated with Mn exhibit oxidative stress-induced signs of inflammation and swelling, which are associated with α-synuclein aggregation, a hallmark of Parkinson's disease (Sarkar et al., 2018[368]). In addition, the process of Mn-induced neuroinflammation involves inducible nitric oxide synthase (iNOS), interleukin-6 (IL-6), and cyclooxygenase-2 (COX-2), and increased expression of nuclear factor erythroid 2-related factor 2 (Nrf2), nuclear factor kappa B (NF-κB), and heme oxygenase-1 (HO-1) (Bahar et al., 2017[20]). The flavonoid quercetin administered to Mn-exposed rats significantly suppressed the levels of oxidative stress markers such as 8-hydroxy-2'-deoxyguanosine (8-OHdG) and activated the immunoreactivity of poly (ADP‒ribose) polymerase (PARP). The inflammatory response triggered by manganese also involves the activation of the mitogen-activated protein kinase (MAPK) signaling pathway in microglia (Jomova et al., 2025[223]; Simunkova et al., 2019[397]).

Chronic exposure of rats to MnSO_4_ for 24 weeks significantly decreased the expression of superoxide dismutase (SOD), catalase, and glutathione peroxidase (GPx) (Cheng et al., 2018[87]). Conversely, the levels of malondialdehyde (MDA) and heat shock protein 70 (Hsp70) increased. The mRNA levels of antiapoptotic Bcl-2, Akt-1, and FoxO3 in manganese-treated rats were decreased, and the level of proapoptotic Bax was increased. In addition, chronic manganese exposure manifests as neuronal apoptosis, upregulated transcription, and translation of Hsp70, as well as activation of the PI3K/Akt signaling pathway, which is important for regulating the cell cycle.

In addition to Parkinson's disease, disturbed Mn homeostasis has been observed in various other neurodegenerative diseases and mental conditions. An examination of 2000 individuals with Alzheimer's disease and mild cognitive impairment revealed significantly decreased levels of manganese compared with those in control groups (Du et al., 2017[127]).

Using immunohistochemical methods, the expression of MnSOD in the hippocampus of patients with Alzheimer's disease and age-matched control subjects without the disease was examined (Marcus et al., 2006[287]). The findings indicate a marked elevation in neuronal Mn-SOD expression in the CA1, CA2/3, and CA4 regions of the hippocampus among subjects with Alzheimer's disease. Additionally, variations in the nonneuronal expression of MnSOD when comparing AD samples to those without the disease were observed. Notably, MnSOD levels in non-AD patients were found to be elevated in astrocytes, whereas in AD samples, this expression was significantly reduced. The impaired activity of MnSOD can be harmful because of the toxicity of ROS, particularly those affecting mitochondrial function. These results indicate that in Alzheimer's disease, there is an effort to mitigate elevated ROS levels by increasing the expression of MnSOD in the pyramidal neurons of the hippocampal formation. Substantial increases in the CA2/3 and CA4 regions of the hippocampus were observed. The CA1 region exhibited a smaller, albeit significant, increase. Notably, the CA1 region is associated with particularly severe neuropathological changes in Alzheimer's disease.

The potential connection between manganese exposure and HD has been the subject of several studies (Aschner et al., 2005[15]). Experimental studies indicate that excessive Mn exposure may exacerbate HD symptoms. This is attributed to iron dysregulation mediated by ferritin and ferroportin proteins. Disrupted iron metabolism hinders the export of manganese, which in turn leads to its accumulation in the central nervous system. Compared with healthy individuals, patients with HD show increased levels of Mn in the bloodstream, which has been associated with degeneration of the basal ganglia, which is typical for Huntington's disease.

Some individuals with epilepsy display reduced whole-blood Mn levels compared with their non-epileptic counterparts. A particular study revealed that individuals with epilepsy of unknown etiology had lower blood Mn levels than those whose epilepsy resulted from trauma, such as head injuries, or other medical conditions (Gonzalez-Reyes et al., 2007[166]). This finding implies a potential genetic relationship between epilepsy and Mn metabolism abnormalities.

**Cancer.** Mn-SOD has emerged as a potential predictive biomarker, as its gene expression varies throughout tumor development-it decreases in the early stages of breast cancer and increases in later stages. The levels of Mn-SOD are directly related to the levels of ROS, mainly superoxide radical anions and hydrogen peroxide, which are involved in signaling pathways that regulate the proliferation and invasiveness of angiogenic cancer cells, thereby contributing to breast tumor growth.

The upregulation of Mn-SOD in cancer cells leads to the continuous production of H_2_O_2_ from the mitochondria, which in turn promotes the activation of AMP-activated protein kinase (AMPK) and facilitates a metabolic transition toward glycolysis. Suppressing Mn-SOD expression or inhibiting AMPK results in the inhibition of this metabolic shift and decreases the survival of transformed cells, highlighting the importance of the Mn-SOD/AMPK pathway in maintaining the bioenergetics of cancer cells, particularly in advanced and aggressive subtypes of breast cancer (Baj et al., 2023[23]; Hart et al., 2015[185]). These findings collectively suggest that Mn-SOD functions as a biomarker for cancer progression and plays a vital role in regulating the metabolism of tumor cells.

Manganese deficiency may play a significant role in carcinogenesis by activating the p53 protein, which triggers apoptosis in cells through mitochondrial pathways (Garrick et al., 2003[156]). Concurrently, this deficiency undermines the antioxidant defense system, which relies predominantly on Mn-SOD. A notable correlation exists between Mn levels and polymorphisms of the Mn-SOD gene, particularly concerning breast cancer risk in perimenopausal women.

Compared with breast and prostate cancers, glioblastoma and melanoma tumors, which are characterized by increased manganese levels, are associated with unfavorable survival rates and suppressed sensitivity to radiotherapy (Doble and Miklos, 2018[120]; Rozenberg et al., 2022[366]). An examination of tissues from Lewis lung carcinoma metastases revealed localized accumulation of manganese in certain primary tumor regions. Several studies have indicated that Mn^2+^ may trigger p53-mediated apoptosis in neuronal cells. Similarly, overexpression of Mn-SOD in colorectal cancer cells can lead to p53-dependent senescence (Behrend et al., 2005[31]).

**Cardiovascular diseases.** The antioxidant capacity of Mn-SOD has been identified as a possible mechanism for decreasing cardiovascular-related illness and death rates. The possible association between manganese exposure and the incidence of cardiovascular diseases in older U.S. adults has been studied (Xiao et al., 2021[458]). In the comprehensive adjusted model, a significant inverse correlation between serum manganese levels and cardiovascular disease in older U.S. adults was identified. Another study reported that manganese can counteract the incidence of cardiovascular disease (Houtman, 1996[199]). Although substantial evidence indicates the positive role of manganese (Mn) in cardiovascular disease, numerous controversies exist. The role of manganese in atherosclerosis, which is the primary cause of the majority of cardiovascular diseases and is characterized by multiple factors, has been the subject of another study (Tang et al., 2024[411]; Nowicki et al., 2021[328]). Furthermore, an increased incidence of abnormal electrocardiograms and notably lower mean diastolic blood pressure than those of the control group were observed in manganese-exposed workers (Jiang and Zheng, 2011[218]). The main characteristics of abnormal electrocardiograms are sinus arrhythmia, sinus tachycardia, sinus bradycardia, left ventricular hypertrophy, and ST-T changes.

**Gastrointestinal tract diseases.** Inflammatory bowel diseases (IBDs), including Crohn's disease and ulcerative colitis, are chronic inflammatory conditions of unknown etiology and pathogenesis that impact the gastrointestinal system in humans (Zanello et al., 2014[471]).

Epidemiological research has revealed a potential link between a greater risk of inflammatory bowel disease and manganese deficiency (Cho et al., 2018[89]). The metal ion transporter SLC39A8 encodes a protein named ZIP8 and is important in the connection between blood manganese concentrations and inflammatory bowel diseases (Choi et al., 2024[90]). Intestinal epithelial cell-specific knockout (Slc39a8-IEC-KO) mice exhibit impaired intestinal absorption and significantly reduced manganese levels in the blood as well as various organs. Single-cell transcriptome analysis of human intestinal tissues confirmed the significant expression of SLC39A8 in both the small and large intestines, highlighting the critical role of these regions in the absorption of metal ions. Slc39a8 is localized at the apical membrane of enterocytes and plays a crucial role in the absorption of manganese in the intestine. Transcriptomic analysis revealed that alkaline ceramidase 1 (ACER1), an enzyme that regulates the levels of ceramides, is a promising therapeutic target for inflammatory bowel diseases associated with SLC39A8. These findings indicate that individuals with inflammatory bowel disease may be at risk for manganese deficiency, which could lead to an increase in the expression of Acer1.

**Renal diseases.** Insufficient intake of dietary manganese has been linked to a heightened risk of developing chronic kidney disease (CKD) in adults who have normal kidney function (Park et al., 2024[337]). The kidney is characterized by high metabolic activity and significant oxidative processes within its cellular mitochondria (Kitada et al., 2020[250]). Mitochondrial oxidative damage and increased oxidative stress are typical in renal tissues. Proximal tubular cells are involved in reabsorbing essential nutrients following glomerular filtration and are especially vulnerable to oxidative stress. The incidence of chronic kidney disease (CKD) and acute kidney injury (AKI) is directly associated with mitochondrial oxidative stress. Dysfunction and suppressed expression of Mn-SOD can be used to explore the impact of oxidative stress on the pathogenesis of renal diseases.

Diabetic kidney disease (DKD) is an independent risk factor for cardiovascular diseases and has been associated with mitochondrial dysfunction as a consequence of impaired Mn-SOD function (Ninomiya et al., 2009). Reduced Mn-SOD activity is documented by increased levels of nitrotyrosine in proximal tubules in renal biopsies from diabetic patients, specifically with increased Mn-SOD Tyr-34 nitration (Xu et al., 2006[461]).

**Pulmonary diseases.** The lungs may serve as a primary target for the toxicity of inhaled manganese dust and nanoscale manganese particles (Barceloux, 1999[28]). Manganese can cause mild to moderate inflammation, manifested by increased cough intensity and bronchitis among workers exposed to increased environmental manganese levels. Elemental manganese at concentrations of 0.2 mM or lower is cytotoxic to normal human lung epithelial cells within a 48-hour period (Pascal and Tessier, 2004[340]). In vitro exposure to 0.25 mM Mn(II) did not result in any observable toxic effects on human lung cancer-derived epithelial cells. Nevertheless, the presence of the metal led to alterations in cell morphology and a significant reduction in the cell growth rate.

Exposure to Mn(II) under in vitro conditions is primarily recognized for its role in promoting the expression of vascular endothelial growth factor (VEGF), a potent mediator and critical marker of angiogenesis, which involves the activation, migration, and proliferation of endothelial cells under various pathological conditions (Gleadle et al., 1995[163]). Exposure to soluble MnCl_2_ enhances the expression of VEGF at the transcriptional level. Mn(II) elevates VEGF promoter activity in a concentration-dependent manner.

The transcriptional activity induced by manganese may occur independently of hypoxia-inducible factors (HIFs) (Bredow et al., 2007[47]). Mn(II) elevated VEGF promoter activity in a concentration-dependent manner. Some similarities between the effects of Mn(II) and those caused by hypoxia or other transition metals have been reported; however, they are not identical. Thus, the pulmonary response to manganese may differ from that elicited by hypoxia.

Exposure to manganese also influences the expression of additional genes linked to the pulmonary vasculature. A greater than twofold increase in vascular endothelial growth factor receptor-1 (VEGFR-1) and a fourfold increase in the level of the endothelial-specific receptor tyrosine kinase for angiopoietins, known as TIE-2, have been reported (Bredow et al., 2007[47]).

Increased levels of VEGF in the lungs are linked to the development of various diseases, including asthma, chronic obstructive pulmonary disease (COPD), pulmonary hypertension, and lung cancer (Papaioannou et al., 2006[336]).

### Manganese-induced ROS and epigenetic alterations

Studies devoted to the epigenetic effects of manganese represent an emerging area of research. Manganese appears to disrupt epigenetic mechanisms, potentially resulting in alterations in developmental programming (Lindner et al., 2022[274]). Frequently recognized epigenetic processes mediated by manganese-induced oxidative stress include DNA methylation, histone modification, and the action of miRNAs at the posttranscriptional level. The interplay between DNA methylation and histone modification regulates the accessibility of transcription factors to the promoter regions of genes.

An analysis was performed to determine the relationships between Mn levels in maternal erythrocytes during the first trimester and differentially methylated positions (DMPs) and regions (DMRs) in cord blood (Bozack et al., 2021[43]). The study revealed that manganese was associated with increased methylation of a specific DNA site, cg02042823, in the gene RNA binding fox-1 homolog 1 (RBFOX1, or A2BP1). In male infants, only two Mn-associated DMPs were identified; however, in female infants, nine DMPs were identified. Notably, no overlap in the DMPs between males and females was observed, suggesting that prenatal Mn exposure may lead to sex-specific alterations in DNA methylation.

The correlations between urinary manganese levels and three DNA methylation-based indicators of biological aging, DNAmAge, GrimAge, and PhenoAge, were examined in elderly men (Nwanaji-Enwerem et al., 2020[329]). The results revealed a significant association with PhenoAge, corresponding to an approximately 10-year increase in DNA methylation-based biological age. Given that manganese is typically excreted through bile rather than through urine, this observation may suggest a partial diversion in Mn excretion to urine due to kidney dysfunction.

Animal models in which pregnant mice receive 800 ppm manganese chloride from the 10th to 20th day of pregnancy have been investigated (Wang et al., 2013[441]). Analysis revealed hypermethylation in the promoter regions of 24 genes within the hippocampal dentate gyrus of male offspring. Exposure to the same concentration of manganese during adulthood resulted in hypermethylation and downregulation of transcripts of genes such as Pvalb, Mid1, Atp1a3, and Nr2f1.

Excessive Mn exposure primarily affects the central nervous system, supporting the development of neurodegenerative disorders such as Parkinson's disease (Tarale et al., 2016[413]). Manganese-induced oxidative stress has an impact on epigenetic regulation in the context of Parkinson's disease. α-Synuclein is a key protein found in substances termed Lewy bodies, which are characteristic markers of Parkinson's disease (Foulds et al., 2010[140]). Manganese exposure has been associated with the overexpression of α-synuclein in neuroblastoma cells (SH-SY5Y), suggesting its role in neuronal apoptosis. α-Synuclein overexpression in manganese-treated PC12 cells is related to the activation of the ERK1/ERK2 MAPK signaling pathway (Cai et al., 2010[64]). Furthermore, hypomethylation of the α-synuclein gene promoter has been linked to its overexpression in Parkinson's disease and related conditions (Frieling et al., 2007[145]). Thus, altered methylation patterns may contribute to the Mn-induced overexpression of α-synuclein.

Parkinson's disease is characterized by the interaction between α-synuclein and DNA methyltransferase (DNMT1), which catalyzes the transfer of a methyl group to DNA. The sequestration of DNA methyltransferase (DNMT1) within the cytoplasm leads to suppressed DNA methylation, affecting genes associated with Parkinson's disease, such as SNCA, SEPW1, and PRKAR2A (Desplats et al., 2011[112]).

Taken together, epigenetic mechanisms, particularly DNA methylation, play crucial roles in neuronal development, and manganese exposure can disrupt these regulatory processes at the epigenetic level (Sun et al., 2011[402]).

## Zinc

Zinc is a low-melting metal and the 30^th^ chemical element in the periodic table of elements with the electron configuration [Ar]3d^10^4s^2^. Zinc is an essential micronutrient and one of the industry's most widely used metals (Charbaji et al., 2021[75]; Jomova et al., 2022[224]). One of the most important industrial applications of zinc is galvanization, which refers to the method of applying a protective layer of zinc to iron or steel to inhibit the formation of rust. The most common oxidation state of zinc in chemical compounds is +2. A limited number of papers have reported zinc compounds where zinc is in the +1 oxidation state. It has been reported that Zn can adopt a +3 oxidation state when it interacts with superelectrophilic clusters (Shang et al., 2022[382]).

Zinc ranks as the second most prevalent micronutrient in the human body, following iron, and is an integral part of more than 2500 proteins that act in the human body (Lim et al., 2013[272]). Zinc serves as an essential trace element within the human body, significantly contributing to a multitude of physiological functions. It is integral to processes including cellular growth and development, metabolic activities, cognitive function, reproductive health, and the overall efficacy of the immune system (Kiouri et al., 2023[248]).

In adult humans, the total zinc content ranges from approximately 1.5 to 2.5 grams, with its presence noted across all organs, tissues, fluids, and secretions. Approximately 80-90% of the total zinc in the body is located in skeletal muscle and bone, whereas smaller amounts are found in the liver, gastrointestinal tract, skin, kidneys, brain, lungs, prostate, and various other organs (Frederickson and Bush, 2001[141]). The concentration of free intracellular Zn(II) is estimated to be as low as 0.5 nM on the basis of measurements obtained from the zinc-specific ^19^F-NMR signal of a fluorinated metal chelating probe (Benters et al., 1997[33]).

The World Health Organization (WHO) reported that approximately one-third of the global population is susceptible to zinc deficiency. Defining and implementing a suitable and sensitive biomarker for the quantitative evaluation of zinc status is awaited.

Zinc is an essential trace element that plays a critical role in gene regulation, enzymatic function, and the integrity of protein structures (Qu et al., 2023[352]). Over 350 enzymes involved in various metabolic and cellular functions within the human body depend on zinc as a cofactor to facilitate their enzymatic activities. Furthermore, zinc is vital for preserving the structural integrity and DNA-binding capabilities of more than 2000 transcription factors.

A low level of zinc may have serious health consequences. Zinc is physiologically interconnected with copper, and excess zinc is usually associated with disrupted copper homeostasis (Maret and Sandstead, 2006[290]). Zinc deficiency can be reversed by dietary supplementation (Walker and Black, 2010[437]).

### Zinc absorption and homeostasis

Cellular zinc homeostasis is characterized by three primary zinc pools: zinc that is bound to proteins, zinc stored within vesicles, and the free zinc pool present in the cytoplasm (Maares and Haase, 2020[283]). Free zinc, which is coordinated by low-molecular-weight organic ligands, is regarded as the biologically active form of the ion. Zinc species undergo redistribution, compartmentalization, or function as signaling molecules (Bozym et al., 2010[44]). The free zinc concentration in the cytoplasm is regulated at nanomolar to picomolar levels. It involves the release of zinc from the cell and its sequestration by zinc transporters into vesicles or its binding to metallothionein (Maret, 2009[289]).

The primary regulatory processes governing zinc homeostasis in humans involve both absorption and excretion, with the small intestine, liver, and pancreas serving crucial functions in its regulation (Krebs, 2000[255]). Zinc absorption in rodents occurs predominantly in the duodenum and ileum of the small intestine; however, the primary site of absorption in humans remains a topic of debate (Maares and Haase, 2020[283]). Zinc is transported from the intestinal lumen to intestinal absorptive cells and enterocytes and subsequently released into the circulation, where it is transported and distributed in the human body by human serum albumin (>50%), α-macroglobulin (~35%), and transferrin (~10%) (Scott and Bradwell, 1983[377]).

**Figure 6:**
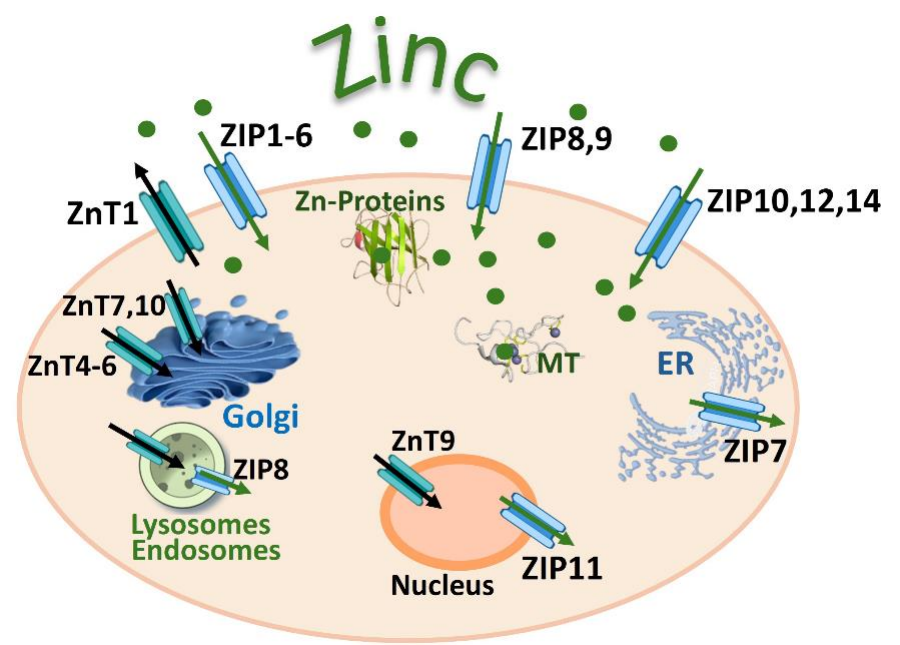
Cellular zinc transport. Zinc transporters (ZnTs), ZIP family of zinc transporters (ZIPs), metallothionein (MT), endoplasmic reticulum (ER), and Golgi apparatus (Golgi).

Cellular zinc homeostasis is intricately controlled by a complex network of proteins, encompassing the solute carrier (SLC) families SLC30 (ZnT) and SLC39 (Zrt- and Irt-like proteins/ZIP), in addition to zinc-binding metallothioneins (MTs) (Chen et al., 2024[76]; Bitirim et al., 2021[38]; Abdo et al., 2021[1]). Earlier studies proposed that the cation transporter of low sensitivity, divalent metal transporter 1 (DMT1), is involved in intestinal zinc uptake (Gunshin et al., 1997[173]). However, the discovery of ZIP family transporters challenged the role of DMT1 in physiological zinc transport. The transport of zinc in the ionic state from the intestinal lumen into enterocytes is achieved by a transmembrane zinc transporter, ZIP4 (encoded by the SLC39A4 gene), and zinc transporter 1, ZNT1 (encoded by SLC30A1). ZIP4 is regulated by dietary zinc at a transcriptional, translational, and post-translational level (Wang et al., 2004[440]). The mechanism of zinc absorption is complemented by basolaterally localized zinc transporters ZIP5 (encoded by SLC39A5) and ZIP14 (encoded by SLC39A14). In addition to the zinc transporters located at the apical and basolateral membranes of enterocytes, ZnT2, ZnT4, ZnT6, and ZnT7 play a role in modulating the cytoplasmic zinc levels within these cells (Palmiter et al., 1996[335]). Interestingly, ZnT2 functions as a vesicular zinc exporter and has been identified in the human in vitro intestinal adenocarcinoma cell line Caco2 (Jou et al., 2010[229]). A simplified scheme of cellular zinc transport is shown in Figure 6(Fig. 6).

The zinc levels in enterocytes affect the expression of metallothioneins, a family of cysteine-rich, low-molecular-weight proteins that serve as an intracellular zinc reservoir. Increased levels of zinc activate metal regulatory transcription factor 1 (MTF-1), which in turn increases the expression of metallothioneins (Andrews, 2000[9]). Increased metallothionein levels serve as a protective barrier against elevated zinc levels in the intestinal lumen; conversely, zinc deficiency is accompanied by the downregulation of metallothionein. Metallothionein affects the rate constant of kinetic transport and may be involved in the efflux of zinc from enterocytes back to the intestinal lumen (Hoadley et al., 1988[192]).

The kinetics of zinc absorption are characterized by carrier-mediated and saturable processes (Yasuno et al., 2012[467]). The fraction of dietary zinc absorbed by the human body ranges from 15-45%. The regulation of zinc absorption reflects an individual's current zinc status, which is related to previous periods of zinc intake. Zinc deficiency is usually accompanied by increased fractional zinc absorption. Oral zinc intake in an aqueous solution is usually more efficient in terms of absorption than that in a solid-state (Maares and Haase, 2020[283]). The physiologically typical amount of zinc intake from a standard meal is approximately 100 µM (Lee et al., 1989[264]). The human body usually contains 2.5 grams of zinc distributed among different organs and cell compartments, with the majority occurring in bones and muscles (~ 80%), followed by the skin and liver (Jackson, 1989[211]).

The amount of zinc absorbed in the intestine is influenced not only by the zinc content in the diet but also by its bioaccessibility (free absorbable zinc concentration) and bioavailability (zinc absorbed by cells) released into the bloodstream (Holst and Williamson, 2008[195]).

### Zinc signaling

The overall concentrations of zinc within cells typically fall between 200 and 300 μM (Maret, 2015[288]). The labile or free zinc pool in eukaryotic cells is reported to be in the nanomolar to picomolar range (Outten and O'Halloran, 2001[332]).

Zinc has been termed the "calcium of the 21st century" because it plays a dual role, acting both as a neurotransmitter for intercellular communication and as an intracellular signaling molecule (Frederickson et al., 2005[142]).

Zinc signaling is derived from three key sources: (i) vesicular exocytosis, (ii) the transport of zinc mediated by specific influx/efflux transporters, and (iii) the interaction/release of zinc with/from metallothioneins (MTs) (Chen et al., 2024[76]).

**Figure 7:**
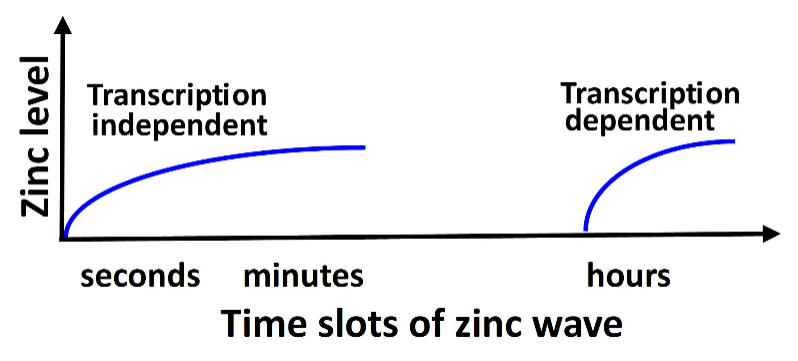
Early and late zinc signaling.

Intracellular zinc acts as a second messenger, with time-dependent concentration fluctuations spanning either early (fast) zinc signaling (EZS) or late zinc signaling (LZS). Early zinc signaling is termed a “zinc wave”, occurs within seconds to minutes, and operates independently of transcription; however, the exact mechanism in the cellular context is not known (Figure 7(Fig. 7)) (Hirano et al., 2008[190]). Early zinc signaling is initiated in the cytoplasmic region around the nucleus (perinucleus space) and is associated with calcium influx (Colvin et al., 2010[93]). In contrast, late zinc signaling persists for several hours and requires the transcription of zinc-based transport proteins. Various extracellular signals, such as cytokines and growth factors, regulate the transcription of the ZIP transporter family of proteins and zinc transport (ZnT) proteins (Yamasaki et al., 2007[462]). Fluctuations in intracellular zinc levels activate downstream molecular targets such as protein kinase C (PKC) and ERK1/2, which play important roles in promoting cell death in a variety of neuronal systems, including neurodegenerative disorders. The transcription factors NF-κB, a serine/threonine-specific calcium/calmodulin-dependent protein kinase II (CaMKII), and protein kinase A (PKA), which belong to the family of serine-threonine kinases whose activity is dependent on the levels of cyclic AMP (cAMP) and other transcription factors, are also activated.

Intracellular zinc may be released in response to oxidative or nitrosative stress, which can lead to the mobilization of zinc from metallothionein (MT), facilitating apoptotic mechanisms (Chen et al., 2024[76]). Increased zinc levels have dual functions in cellular processes. Physiological zinc levels are associated with interference with numerous signaling pathways that impact cell proliferation and differentiation and the development of the embryonal CNS via the signal transducer and activator of transcription 1 (STAT1) and signal transducer and activator of transcription 3 (STAT3) pathways (Supasai et al., 2017[405]). In addition, zinc may be involved in hematopoiesis more profoundly than iron. Zinc is an important modulator of immune functions, and the development of a specialized type of immune cell, such as T cells, plays a role in protection against infections and cancer. Despite rather low levels of intracellular zinc, its signaling capacity is satisfactory for maintaining important physiological functions. On the other hand, high, nonphysiological levels of intracellular zinc, specifically derived from mitochondria, may disrupt its integrity and trigger apoptotic pathways (Chen et al., 2019[77]).

Extracellular zinc is an important mediator of endocrine (secreting hormones), autocrine (secreting a hormone/chemical messenger), and paracrine (serving signals to adjacent cells) signaling (Millward, 2017[310]). Zinc is involved in neuronal excitation and regulation of the human brain's primary glutamate receptor, N-methyl-D-aspartate (NMDA) (Anderson et al., 2015[8]), γ-aminobutyric acid A (GABAA) receptors (Carver et al., 2016[69]), voltage-dependent calcium channels (VDCCs) (Michelotti et al., 2020[308]) and others. Variations in zinc levels in different brain regions are correlated with neurotransmitter activity and the physiological mechanisms of long memory (Ren et al., 2020[360]). A combination of advanced techniques, such as transmission electron microscopy (TEM) combined with single-cell amperometry (SCA), revealed that zinc alters, among other features, neurotransmitter release through modulation of presynaptic plasticity (Ren et al., 2020[360]).

### Zinc and oxidative stress

Unlike redox-active copper and iron, zinc, as a redox-inactive metal, is not involved in electron transfer reactions (Valko et al., 2005[430]). The mechanism of the antioxidant action of zinc can be acute or chronic. The acute antioxidant role of zinc was first suggested in the late 1980s and is characterized by two primary mechanisms: (i) the prevention or antagonism of redox metal-induced ROS formation and oxidative stress and (ii) the protection of the sulfhydryl groups of proteins and enzymes against ROS attack (Bray et al., 1990[46]).

(i) Oxidative modifications of proteins primarily occur at redox metal binding sites, where their catalytic activity via the Fenton reaction results in the formation of damaging ^•^OHs (Stadtman, 1990[399]). The potential protective mechanism involves the extraction or "pulling" of the redox-active metal from its binding site by a high-affinity ligand or chelator. The second mechanism is characterized by a "pushing" effect, where a chemically similar but redox-inactive metal (for example, zinc, which replaces isostructural copper) displaces the redox-active metal from its binding site. The displaced redox metal can subsequently be eliminated from the cell, thereby decreasing its bioavailability for participation in ^•^OH generation via the Fenton reaction. This “antagonism” mechanism was confirmed by EPR spin trapping of ^•^OH generated from iron and cysteine in the presence of zinc, indicating that the competitive interaction between the two metals and the thiol amino acid disrupts the electron transfer to oxygen (Searle and Tomasi, 1982[378]).

Furthermore, zinc has been shown to inhibit iron-mediated peroxidation of erythrocyte membranes induced by xanthine and xanthine oxidase (Girotti et al., 1985[161]). The antagonistic effects of zinc on radical formation have also been documented in various systems, including copper-iron ascorbate-induced DNA strand breaks (Harel and Chevion, 1991[183]), superoxide and ^•^OH production from xanthine oxidase and NADPH oxidase (Powell, 2000[350]), and Fe(III)-ascorbate-induced methemoglobin formation in red blood cells (Shinar et al., 1989[391]), among other systems.

(ii) The stabilization function of zinc on the sulfhydryl groups of specific enzymes is manifested by a protective mechanism against oxidative reactions. Notably, δ-aminolevulinate dehydratase has been extensively studied, as it facilitates the conversion of two molecules of δ-aminolevulinic acid into the pyrrole porphobilinogen. In light of these investigations, three structural models that may describe the stabilization of sulfhydryl groups have been proposed (Gibbs et al., 1985[159]). The first model assumes a direct interaction between zinc ions and the sulfhydryl groups, whereas the second model assumes that zinc binds to a site adjacent to the sulfhydryl groups. The third model is based on the assumption that zinc interacts with a different site on the protein, leading to a conformational alteration of the protein structure. Each of these proposed models indicates a reduction in the reactivity of the sulfhydryl groups. Research has demonstrated that zinc protects various sulfhydryl-containing proteins, including dihydroorotases (Kelly et al., 1986[243]), DNA zinc-binding proteins (commonly referred to as zinc fingers) (Klug and Rhodes, 1987[251]), alanyl tRNA synthetases (Wu et al., 1994[456]), class I tRNA synthetases (Landro and Schimmel, 1993[261]), 5-enolpyruvylshikimate-3-phosphate synthases (Padgette et al., 1988[333]), *E. coli* DNA topoisomerase I (Tsedinh and Beransteed, 1988[423]), and protein farnesyltransferases (Fu et al., 1996[146]).

Metallothioneins are composed of 60 to 68 amino acid residues, of which approximately 25 to 30% are cysteine residues, and have the capacity to effectively bind zinc and serve as a link between the cellular zinc level and the cellular redox state. Under conditions of elevated oxidative stress, an altered cellular redox state can trigger the release of zinc from metallothioneins through sulfide/disulfide exchange mechanisms (Jiang et al., 1998[216]). Long-term zinc supplementation can have several beneficial health effects attributed to the induction of various antioxidant molecules, including metallothioneins, which are the most prominent (Powell, 2000[350]). Metallothioneins can also scavenge ROS through thiol groups in cysteine residues; for example, metallothionein-2, in response to oxidative stress, is elevated and has been shown to possess a scavenging capacity for hydroxyl (^•^OH) and peroxyl (ROO^•^) radicals that is significantly greater than that of GSH.

Metallothioneins significantly contribute to the structural stability of enzymes such as nitric oxide synthase (NOS) (Pluth et al, 2011[346]), matrix metalloproteinase-9 (MMP-9) (Rowsell et al., 2022[365]), and Cu,Zn-superoxide dismutase (Cu,Zn-SOD, SOD1) (Choi et al., 2018[91]). Notably, metallothioneins become particularly active when the reduced form of glutathione (GSH) is unavailable, effectively scavenging free radicals via the Zn-MT redox mechanism. Metallothioneins have been reported to play a role in modulating ERK phosphorylation and regulating ROS levels through heme oxygenase-1 (HO-1) (Qu and Waalkes, 2013[351]). The effectiveness of metallothionein-3 (known as growth inhibitory factor) in reducing ROS has been notably associated with its affinity for metal binding (Koh and Lee, 2020[252]).

Zinc deficiency is usually accompanied by elevated levels of oxidative damage in tissues, manifested by increased oxidation of lipids, proteins, and DNA. Animal studies have demonstrated that prolonged zinc deprivation increases an organism's vulnerability to injuries induced by oxidative stress. The adverse effects of zinc deficiency, particularly in relation to the production of reactive oxygen species (ROS), have been evidenced by instances of hyperoxic lung damage (Taylor et al., 1988[415]), the formation of conjugated dienes and malondialdehyde in liver microsomes (Sullivan et al., 1980[401]), and the generation of carbon-centered free radicals in lung microsomes. Rats subjected to a low-zinc diet presented reduced activity of glutamine synthetase, diminished production of Fe(II)-stimulated 2-thiobarbituric acid-reactive substances (TBARS), elevated concentrations of protein carbonyls, and increased levels of the biomarker 8-oxo-2'-deoxyguanosine (8-OH-dG) (Oteiza et al., 1995[331]). Additionally, zinc deficiency has been associated with induced lipid peroxidation in liver microsomes and mitochondria (Burke and Fenton, 1985[58]), increased sensitivity to copper-mediated lipoprotein oxidation (DiSilvestro et al., 1997[116]), and the occurrence of galactosamine-induced hepatitis in rats (Parson and DiSilvestro, 1994[338]).

Zinc has the capacity to preserve the integrity of the blood‒brain barrier (BBB), a highly specialized semipermeable and selective vascular system within the central nervous system of most vertebrates (Noseworthy and Bray, 2000[327]). The membrane of the BBB is highly rich in polyunsaturated fatty acids, which makes it susceptible to ROS attack. Zinc deficiency, which is typical for bacterial and viral infections, significantly increases the permeability of the BBB. Thus, the normal function of the BBB and sufficient protection against ROS attack require an adequate supply of zinc. Such antioxidant protection of the BBB against oxidative stress may help divert the onset of neurodegenerative disorders such as dementia, Alzheimer's disease, and other diseases.

### Zinc, the immune system, and inflammation

Zinc is recognized for its pivotal function within the immune system, with individuals lacking sufficient zinc exhibiting heightened vulnerability to numerous pathogens (Shankar and Prasad, 1998[383]). The immunological processes through which zinc influences this increased susceptibility to infections have been the subject of investigation for several decades. The proper function of the immune system is closely associated with the physiological levels of zinc. The immune response can be categorized into two primary components: innate immunity and adaptive immunity (Bonaventura et al., 2015[40]).

The initial response to pathogens is associated with the innate immune system, specifically, polymorphonuclear cells (PMNs), macrophages, and natural killer (NK) cells. In contrast, specialized cells known as T and B lymphocytes mediate the adaptive immune response. B lymphocytes contribute to the humoral immune response by producing antibodies that target specific antigens. In contrast, T lymphocytes facilitate cell-mediated immunity by activating other immune cells (T helper lymphocytes) and generating cytotoxic granules in cytotoxic T lymphocytes (Bonaventura et al., 2015[40]). The interface between innate and adaptive immunity is mediated by dendritic cells (DCs). Zn is directly implicated in the innate immune response. Zinc deficiency results in reduced chemotaxis, which is important for enabling polymorphonuclear neutrophils (PMNs) to reach sites of infection and inflammation (Ibs and Rink, 2003[207]). Conversely, high zinc levels (> 450 μM) may induce PMN chemotaxis under in vitro conditions (Sheikh et al., 2010[387]). The impact of zinc on phagocytosis is mediated by zinc proteins such as early endosomal antigen 1 (EEA1), which are primarily composed of α-helical protein regions bordered by cysteine-rich, metal-binding "finger" motifs.

Normal zinc levels are very important in the deactivation of pathogens. Both anomalies in zinc concentration, excess, and deficiency can impede the function of nicotinamide adenine dinucleotide phosphate (NADPH oxidase), a membrane-bound enzyme that produces superoxide radical anions crucial for the elimination of pathogens (Hasegawa et al., 2000[186]).

The maturation of dendritic cells (DCs) plays a crucial role in the formation of T-cell responses and the establishment of immune tolerance. Lipopolysaccharide (LPS)--induced maturation of dendritic cells is dependent on zinc; notably, the expression of the Zn transporters ZIP6 and ZIP10 is downregulated, and the expression of zinc transporters (ZnTs) is upregulated. LPS stimulation is known to suppress the level of zinc in dendritic cells and trigger the expression of zinc-dependent major histocompatibility complex class II (MHC class II) (Huang and Tepaamorndech, 2013[203]).

The development and function of T cells are very sensitive to zinc deficiency. A lack of zinc is characterized by enhanced apoptosis of T cells, a significant reduction in “effective” and “potential” T cells in mice, and a disturbed balance between the different T-cell subsets (King et al., 2005[247]).

Zinc activates T cells by promoting the autophosphorylation of the tyrosine-protein kinase Lck through its interaction with the cytoplasmic domains of CD4 (helper T cells) and CD8 (killer T cells). Various functions of T cells are regulated by the interaction of Zn-MT through the phosphorylation of signal transducer and activator of transcription 1 (STAT1) and signal transducer and activator of transcription 3 (STAT3) transcription factors. This mechanism is important for the survival of T cells (Lin et al., 1998[273]).

B cells (a type of white blood cell called lymphocytes) are less sensitive to alterations in zinc levels than are T cells and protect the organism from infection by producing antibodies. Zn deficiency affects the development of B cells and, consequently, the production of antibodies (DePasquale-Jardieu et al., 1984[111]).

Zinc deficiency affects the secretion of cytokines by immune cells, which may play a key role in the inflammatory response. This process is accompanied by the secretion of the proinflammatory cytokine IL-1β. Zinc supplementation can reverse the inflammatory response (Dore-Duffy et al., 1990[125]). Reduced zinc levels correlate with increased secretion of IL-8, TNF-α, and IL-6. In addition, the reaction characterized by IL-6 and IL-2 stimulation caused by zinc deficiency enhances the proliferative responses of T and B lymphocytes. Conversely, the signaling of IL-4 is negatively affected, leading to a compromised immune response (Gruber et al., 2013[169]).

The above discussion confirms that zinc significantly affects various aspects of the innate and adaptive immune systems and has a profound impact on the immune response.

### Zinc and human disease

#### Alzheimer's disease

The hallmark of Alzheimer's disease (AD), the amyloid-β hypothesis, has been discussed in the preceding chapters devoted to iron and copper. Under normal physiological conditions, amyloid-β is produced in small amounts from its precursor protein (APP) (Leissring et al., 2008[266]). Zinc metalloproteinases effectively cleave APP at the amino side of hydrophobic residues at a high rate. One of the most effective cleavage enzymes of APP is the endopeptidase neprilysin (Leissring et al., 2008[266]).

The role of nonredox zinc in the pathogenesis of AD remains controversial (Shippy et al., 2024[392]). It has been suggested that at micromolar concentrations, zinc may inhibit Aβ-induced toxicity; however, the precise mechanisms underlying the protective effects of zinc against Aβ toxicity are not fully understood.

The attempt to quantify zinc levels in relation to neurodegenerative diseases, including Alzheimer's disease, has led to controversial results. Zinc is essential in the context of chronic age-related diseases, as the intestinal absorption of zinc tends to decrease with age (Coudray et al., 2006[99]). Additionally, serum zinc concentrations decline with age, with a more significant reduction observed in patients with Alzheimer's disease (AD) than in healthy individuals of similar age (Brewer, 2012[52]). Deficiencies in zinc can lead to disruptions in cell signaling and activation, thereby exacerbating the proinflammatory response (Wong et al., 2015[454]).

Conversely, numerous studies have indicated that zinc accumulation in the brain is a prominent characteristic of AD, as evidenced by postmortem examinations (Religa et al., 2006[359]; Deibel et al., 1996[110]). It has been reported that zinc levels in the cortical tissue of AD patients are more than double those in healthy controls. Zinc concentrations mimic the tissue levels of amyloid-beta (Aβ) (Religa et al., 2006[359]). Further work on standardization protocols for sample collection and quantitative zinc evaluation via sensitive experimental techniques is essential.

Many studies have confirmed that not only the levels of zinc but also the interplay between isostructural zinc and copper is particularly relevant in the context of AD (Cuajungco et al., 2000[103]). The protective role of zinc has been attributed to its potential to compete with copper (and iron) for binding to amyloid-β. Zinc coordinated with residues of amyloid-β disturbs its conformational state to such an extent that copper ions are unable to access its metal-binding sites. This preventive activity of zinc to saturate the copper-binding sites of amyloid-β may inhibit the copper-amyloid-β-catalyzed formation of H_2_O_2_ via the Fenton reaction, producing ROS, including reactive ^•^OH, and causing neuronal toxicity and oxidative damage. Conversely, increased levels of oxidative/nitrosative stress originating from both endogenous and exogenous sources can disrupt the normal metabolism of amyloid-β and trigger excessive release of vesicular zinc (Cuajungco et al., 1998[104]).

While low-to-physiological concentrations of zinc are protective against amyloid-β toxicity, high concentrations of zinc released by oxidative agents may initiate neuronal death, either independently or in conjunction with the toxic effects of amyloid-β. These findings are consistent with research indicating that elevated zinc levels can cause amyloid-β to precipitate across a relatively broad pH range (6-8) (Cuajungco et al., 2003[102]). Furthermore, zinc binding has been found to preserve the α-helical structure of Aβ(1-40) in a highly ordered conformation.

Thus, under normal physiological conditions, a delicate equilibrium exists among zinc, copper, and amyloid-β metabolism. However, oxidative/nitrosative stress and inflammatory conditions, which are typical for neurodegenerative disorders such as Alzheimer's disease, may disturb this fragile balance between metals and consequently disrupt zinc homeostasis, resulting in the uncontrolled release of zinc and amyloid deposition. The uncontrolled accumulation of either zinc or amyloid-β could contribute to zinc-induced and Aβ-mediated oxidative stress and cytotoxicity.

Numerous investigations have revealed that disturbances in zinc homeostasis within the brain lead to τ aggregation and ultimately facilitate the formation of neurofibrillary tangles (NFTs) (An et al., 2005[7]; Li et al., 2023[270]). Zinc is known to activate various kinases associated with the hyperphosphorylation of τ, including extracellular regulated protein kinase 1/2 (ERK1/2), c-Jun N-terminal kinase (JNK), and glycogen synthase kinase-3β (GSK-3β), along with other Src-dependent signaling pathways (Wang et al., 2020[442]). Additionally, zinc inhibits major τ phosphatases, which is manifested by promoted τ-hyperfosforylation. The aggregation of τ supports the binding of zinc to C- and N-terminal τ regions (La Rocca et al., 2022[258]).

Ferroptosis is a relatively recently identified distinct form of cell death characterized by significant iron accumulation and lipid peroxidation (Dixon, 2017[117]). Characteristic features of ferroptosis include the generation of ROS, the inactivation of glutathione peroxidase 4 (GPX4), alterations in cellular morphology, the iron-mediated accumulation of lipid peroxides, and a reduction in glutathione (GHS) levels. Since amyloid-β plaques contain increased levels of iron and zinc, their interaction in the context of the pathogenesis of AD represents a compelling area for further research. Research has extensively examined the role of ferroptosis and zinc in Alzheimer's disease (AD), revealing that the aforementioned features are present in the brains of individuals afflicted with this condition. The cytotoxic effects of intracellular amyloid-beta (Aβ) are known to facilitate ferroptosis in neuronal cells, and various studies indicate that microglia may also be vulnerable to cell death mediated by ferroptosis. Notably, microglia have been implicated in driving neurodegeneration through ferroptosis. Zinc has been found to directly facilitate microglial activation, as evidenced by increased expression of the glycoprotein F4/80, increased nitric oxide (NO^•^) production, and elevated activity of NF-κB (Kauppinen et al., 2008[236]). The application of ZnCl_2_ significantly increases the release of proinflammatory mediators, such as TNF-α, IL-6, and IL-1β, in microglia stimulated with lipopolysaccharide (LPS), suggesting that zinc may sensitize microglia to release these mediators by modulating ROS levels (Higashi et al., 2017[188]). Zinc can prevent ferroptosis by activating the nuclear factor erythroid-2-related factor 2/heme oxygenase 1 (Nrf2/HO1) and GPX4 signaling pathways. In addition, zinc plays a significant role in modulating iron levels in the context of AD, as it has been shown to inhibit the ferroxidase activity of amyloid precursor protein (APP).

#### Parkinson's disease

The levels of zinc in the tissues of patients with Parkinson's disease (PD) have been reported to vary in consistency, leading to ambiguity regarding their status (Ayton et al., 2013[17]). Earlier studies indicated an increase in zinc concentrations within the substantia nigra (SN) of PD patients (Dexter et al., 1989[114]). However, this finding was not corroborated by later research employing X-ray microanalysis techniques (Hirsch et al., 1991[191]).

Levodopa is a dopamine precursor used in Parkinson's disease therapy. A recently conducted study revealed that serum zinc deficiency in Parkinson's disease patients with levodopa-induced dyskinesia (79.6 μg/L) was more pronounced than that in patients without levodopa-induced dyskinesia (88.2 μg/L) (Kim et al., 2023[246]). This research indicates that a deficiency in serum zinc may pose a risk for levodopa-induced dyskinesia in patients with Parkinson's disease who have not yet received treatment.

The concentration of zinc in the cerebrospinal fluid (CSF) of patients with Parkinson's disease has been examined; however, conflicting results have been reported. In one report (Molina et al., 1998[312]), a reduction of approximately 40% in CSF zinc levels among PD patients was reported, whereas another work (Hozumi et al., 2011[200]) reported an almost threefold increase in CSF zinc levels in the same population.

Changes in intracellular zinc homeostasis have emerged as significant factors in the pathogenesis of Parkinson's disease. Both a deficiency and an excess of intracellular zinc have been associated with the onset of the disease, although substantial evidence tends to support the latter mechanism (Sikora and Ouagazzal, 2021[393]). In addition to its critical involvement in numerous cellular processes, Zn^2+^ functions as a synaptic transmitter within the brain. Recent findings suggest that disruptions in vesicular (or synaptic) Zn^2+^ signaling within the basal ganglia may also play a role in the development of this disease.

The brain's response to neurotoxins expressed through changes in zinc levels has been studied. Changes in the concentrations of several redox metals and non-redox zinc have been analyzed in various brain regions of animal models of Parkinson's disease (Lee et al., 2009[265]). In another study, mice received intraperitoneal injections of 1-methyl-4-phenyl-1,2,3,6-tetrahydropyridine (MPTP), an organic precursor of the neurotoxin MPP+ (Tarohda et al., 2005[414]). Dopaminergic neurons were assessed for zinc accumulation and signs of apoptosis. The use of zinc-specific fluorescent dyes in the substantia nigra pars compacta revealed that all degenerating neurons exhibited apoptotic cell death and elevated levels of cytosolic labile zinc.

Taken together, these findings indicate that endogenous Zn^2+^ plays a significant role in the pathophysiology of Parkinson's disease. Nevertheless, the role of this metal cation is quite intricate, as intracellular Zn^2+^ has been associated with both beneficial and harmful effects in the context of this disorder (Sikora and Ouagazzal, 2021[393]). The beneficial effects are primarily related to the protective role of Zn^2+^ against oxidative stress, which is mediated through its impact on the activity of antioxidant enzymes, such as Cu, Zn-SOD, and various signaling pathways. Conversely, the detrimental effects of zinc are linked to oxidative stress resulting from an overload of intracellular Zn^2+^. In addition to changes in intracellular zinc levels, recent findings suggest that impairments in synaptic Zn^2+^ signaling may also play a role in the development of PD.

#### Zinc and cancer

Zinc plays a crucial role in numerous enzymatic reactions and physiological functions, and its deficiency has been associated with various diseases, particularly cancer (Sugimoto et al., 2024[400]). In recent years, there has been a surge of meta-analyses and reviews examining zinc concentrations across different cancer types, alongside numerous primary studies that establish a correlation between reduced zinc levels and specific cancer types.

**Esophageal cancer** has been extensively studied in relation to zinc levels and cancer incidence. Patients in China with esophageal cancer reportedly have lower zinc intake than healthy individuals do (Lu et al., 2006[281]). In agreement with this study, an ecological analysis revealed that African countries with a higher incidence of esophageal cancer had a lower zinc supply than those with lower incidence rates (Schaafsma et al., 2017[372]).

Animal experiments revealed that zinc deficiency leads to increased cell proliferation, modifications in mRNA and microRNA gene expression, and the promotion of esophageal squamous cell carcinoma (ESCC). (Fong et al., 2017[137]) The esophagus of Zn-deficient rats presented a unique metabolic profile characterized by a > 150-fold decrease in glucose and a 1.7-fold increase in lactic acid, indicative of aerobic glycolysis, commonly referred to as the "Warburg effect," which is a signature of cancerous cells. Upon Zn replenishment, there was a rapid increase in glucose levels, normalization of deregulated metabolites to baseline levels, and reversal of the hyperplastic phenotype. Further research revealed that zinc deficiency reprograms glucose metabolism, supporting aberrant cell proliferation by modulating the expression of miR-143 and its target, hexokinase 2 (HK2).

**Zinc and breast cancer. **There are several types of breast cancer. Invasive breast cancer is the most common type of breast cancer, followed by invasive lobular/ductal carcinoma. Breast cancer cells that have estrogen receptors are called ER-positive (or ER+) cancers and may need estrogen to grow.

Initial trials have demonstrated a strong association between low zinc concentrations and the onset and progression of breast cancer. A cross-sectional study carried out in Nigeria in 2015 revealed that the ratio of copper to zinc was markedly elevated in breast cancer patients relative to healthy individuals and that the zinc levels in venous blood were decreased among those with breast cancer (Adeoti et al., 2015[3]). Conversely, an in situ analysis indicated that zinc levels were significantly lower in patients with invasive ductal carcinoma than in those with normal ductal epithelium (Costello et al., 2016[98]).

A recent meta-analysis examined the concentrations of zinc in breast tissue, plasma, serum, and hair samples from individuals diagnosed with breast cancer (Jouybari et al., 2019[230]). The findings indicated that patients with breast cancer presented markedly lower zinc levels in both the blood and hair samples than the control patients did, whereas the zinc concentrations were significantly elevated in the tumor tissues. Another analysis assessed serum copper and zinc levels in breast cancer patients (Feng et al., 2020[134]). The analysis revealed that higher serum copper levels, an increased copper/zinc ratio, and lower serum zinc levels were significantly correlated with a heightened risk of developing breast cancer. The collective findings from meta-analyses and clinical studies enhance the current understanding of the relationship between breast cancer and zinc deficiency (Sugimoto et al., 2024[400]).

The zinc transporter SLC39A7 (ZIP7) is activated in estrogen receptor-positive (ER+) breast cancer cells through seryl phosphorylation, which in turn stimulates pathways that are implicated in cancer progression (Taylor et al., 2012[417]; Ziliotto et al., 2018[483]). ZIP7 activation contributes to the development of resistance against a frequently used treatment for estrogen receptor (ER)-positive breast cancer, tamoxifen. In addition, ZIP7 may function as a suitable biomarker for breast cancer.

Zinc transporter ZIP6 (LIV-1) is a protein encoded by the SLC39A6 gene (Baltaci and Yuce, 2018[25]) that plays a role in both ER+ and metastatic breast cancers (Taylor, 2008[416]). ZIP6 has been found to be upregulated in ER+ breast cancers; however, in primary breast tumors, ZIP6 is notably downregulated. This reduction in ZIP6 expression is associated with increased resistance to hypoxic conditions and facilitates the process of epithelial‒mesenchymal transition (EMT), which is closely related to cancer progression and metastasis (Takatani-Nakase, 2018[408]). Thus, the function of ZIP6 in ER+ breast cancer is dependent on the disease stage and may serve as a reliable biomarker in this type of cancer (Bendellaa et al., 2024[32]).

Zinc metal ions play crucial roles in ferroptosis in renal and breast cancers (Chen et al., 2021[83]). Moreover, Zn concentrations significantly influence sensitivity to ferroptosis; specifically, the zinc transporter ZIP7 was found to increase cytosolic Zn levels, while its inhibition resulted in endoplasmic reticulum (ER) stress responses, increased expression of homocysteine-responsive endoplasmic reticulum-resident ubiquitin-like domain member 1 protein encoded by the *HERPUD1* gene. HERPUD1 may be a good target for suppressing tumorigenesis in breast cancer cells, and conferred protection against ferroptosis (Chen et al., 2021[83]). Zinc finger proteins play pivotal roles in cancer development and are involved in epithelial‒mesenchymal transition (EMT), intratumoral angiogenesis, and cell proliferation and migration in breast cancer (Bassey--Archibong et al., 2016[30]). The activation of aerobic glycolysis by zinc finger E-box binding homeobox 1 (ZEB1) facilitates the polarization of M2-like tumor-associated macrophages, ultimately promoting breast cancer cell growth, metastasis, and chemoresistance. ZEB1 may also serve as a potential therapeutic target for advanced breast cancer (Jiang et al., 2022[214]). Additionally, insights into the mechanism of zinc finger binding to DNA enable the design of proteins that can specifically bind to predetermined DNA sequences (Qu et al., 2023[352]).

**Zinc and lung cancer.** The correlation between zinc levels and lung cancer has been thoroughly examined. One study reported that serum zinc concentrations were markedly lower in lung cancer patients than in control individuals in both European and Asian demographics (Wang et al., 2019[445]). Another analysis reported that individuals with lung cancer presented a significantly elevated serum copper/zinc ratio, indicative of reduced zinc levels, compared with healthy individuals and those with benign lung conditions (Zhang et al., 2022[476]); notably, this ratio was found to be significantly greater in patients diagnosed with advanced lung cancer than in those with early-stage disease. Interestingly, elevated plasma zinc levels may lower the risk of lung cancer, potentially by delaying telomere shortening and influencing the expression of certain oncogenes; the authors propose that zinc could serve as a preventive measure against lung cancer (Bai et al., 2019[21]). Collectively, these studies underscore the role of zinc deficiency in the onset and progression of lung cancer (Sugimoto et al., 2024[400]).

The exact role of zinc in lung cancer has not yet been completely elucidated, as there are inconsistent expression levels of zinc transporters, specifically ZIPs and ZnTs, in various lung cancer cell lines and tumors (Huang et al., 2019[202]). In general, ZnT transporters are downregulated, whereas ZIP transporters are upregulated across various types of lung cancer (Bai et al., 2019[21]). Furthermore, in lung tumor tissues, multiple genes are overexpressed, suggesting that zinc may play a protective role against the development of lung cancer (Bendellaa et al., 2024[32]).

In the most common type, non-small cell lung cancer (NSCLC), there is potential overexpression of ZIP4. ZIP4 is also an essential regulator of the Snail-N-cadherin signaling axis (Jiang et al., 2021[217]), promoting NSCLC progression and metastasis, making it a novel predictive marker and thereby aiding in the development of more precise personalized treatment strategies. ZIP4 levels are positively correlated with increased invasiveness and decreased survival rates among patients (Liu et al., 2013[277]).

**Zinc and prostate cancer. **The human prostate contains unusually high amounts of zinc (Costello et al., 2016[98]), which promotes the formation of prostatic fluid and inhibits the oxidation of citrate (Costello et al., 2006[97]). Prostate cancer cells exhibit a diminished capacity to accumulate Zn, most likely due to the generation of oxidized citrate, which hampers Zn uptake and can induce cell cycle arrest and apoptosis in prostate cancer cells, thereby playing a role in the suppression of malignant cell proliferation (To et al., 2020[421]). The impact of Zn on prostate cancer has yielded mixed findings; the predominant body of evidence suggests that lower Zn levels correlate with a poorer prognosis (Bendellaa et al., 2024[32]).

The ZIP1 protein is responsible for the active transport of Zn into prostate cells, and its expression has been reported to be significantly reduced in prostate cancer (Costello et al., 2016[98]). In addition, a transmembrane transporter, ZIP4, is significantly downregulated in prostate carcinoma and could be a sensitive biomarker for the early detection of prostate cancer, along with metallothioneins, which are zinc-binding proteins typically elevated in prostate cancer cells. Metallothioneins are known to bind zinc effectively, and their serum levels are typically elevated and may serve as tumor markers in prostate cancer.

#### Zinc and cardiovascular disorders

The impact of zinc deficiency on the pathophysiological mechanisms of cardiovascular diseases (CVDs) has been the subject of many studies (Nazari et al., 2023[322]). A meta-analysis investigating zinc supplementation revealed that both the dose and duration of zinc intake significantly suppressed the risk of cardiovascular diseases. The analysis revealed that prolonged supplementation at lower doses was more beneficial than shorter durations (< 12 weeks) with higher doses (> 25 mg/day) (Pompano et al., 2021[348]). Furthermore, another meta-analysis focusing on randomized controlled trials indicated that zinc supplementation positively affects serum zinc levels in prepubertal children [Brown et al., 2002[54]]. Additionally, numerous other studies have established a correlation between serum zinc concentrations and CVD risk, with particularly low serum zinc levels linked to an elevated risk of developing cardiovascular diseases (Hara et al., 2023[182]).

Numerous studies have revealed the beneficial role of zinc in cardiovascular disease, substantiated by mitigated ROS-induced oxidative stress and inflammation, two key molecular mechanisms associated with the incidence of cardiovascular diseases. The NF-κB signaling pathway activated by ROS triggers the expression of proinflammatory cytokines, including IL-1β, IL-6, IL-8, and TNF-α, as well as inducible isoforms, specifically inducible nitric oxide synthase (iNOS) and cyclooxygenase-2 (COX2), identified during inflammatory conditions, resulting in the synthesis of large quantities of proinflammatory and cytotoxic nitric oxide (NO^•^) and prostaglandins (PGs), respectively (Hara et al., 2023[182]).

Zinc forms a complex with metallothionein and serves as a significant inducer. The mechanism through which Zn induces metallothionein involves zinc-dependent metal-responsive transcription factor 1 (MTF-1). MTF-1 stimulates metallothionein gene expression, thereby protecting against oxidative stress (Marreiro et al., 2017[291]). Under conditions of oxidative stress, zinc is released from the Zn-metallothionein complex. Zn is also an essential structural element of the antioxidant enzyme superoxide dismutase (Cu,Zn-SOD), which catalyzes the dismutation of O_2_^•^-into H_2_O_2_ and O_2_. Zinc modulates the expression of glutamate cysteine ligase, referred to as γ-glutamylcysteine synthetase (γ-GCS), which is the enzyme that catalyzes the initial and rate-limiting step in the biosynthesis of the tripeptide glutathione (GSH), one of the most efficient intracellular antioxidants. This enzyme is essential for preserving the intracellular thiol redox balance and facilitating detoxification mechanisms.

The importance of metallothionein in mice has been demonstrated in myocardial contractility (the innate ability of the myocardium to contract), which has been attributed to oxidative stress and mitochondrial impairment. Metallothionein expression induced by Zn prevents myocardial contractility in all accompanying pathologies, such as increased ROS production and mitochondrial damage (Cai et al., 2006[63]).

Diabetes-mediated oxidative stress causes cardiac apoptosis as a result of a hyperglycemia-activated mitochondrial pathway involving cytochrome c and caspase-3. Owing to its strong antioxidant properties, metallothionein suppressed ROS formation and mitochondrial damage and inhibited the progression of diabetic cardiomyopathy (Hu et al., 2013[201]). These and other studies confirmed that metallothionein is an important component of the antioxidant network that protects cardiac functions by alleviating oxidative stress and inflammation. There is no doubt that zinc is associated with the beneficial effects of metallothionein; however, understanding the exact role of zinc in this mechanism requires further studies (Hara et al., 2023[182]).

#### Zinc and schizophrenia

Zinc is likely to play a significant role in the pathophysiology of various mental disorders because of its involvement in multiple biological processes (Kambe et al., 2015[233]). The relationship between zinc dysregulation and psychiatric disorders is becoming increasingly convincing. Zinc is localized mainly in glutamatergic neurons, where it acts as an inhibitory modulator of glutamate *N*-methyl-D-aspartate receptor (NMDAR), which is a calcium-gated channel that coordinates with G protein-coupled receptors to maintain synaptic transmission (Frederickson et al., 2000[143]).

Zinc is present at the highest concentration within the brain's limbic system, where it is secreted in vesicles among a network of glutamatergic zinc-enriched neurons found at hippocampal synapses. Under neurodegenerative and psychiatric conditions such as schizophrenia, presynaptic neurons and astrocytes release excessive amounts of zinc, leading to neuronal death through the activation of microglia and NADPH oxidase and the subsequent production of ROS in neurons (Kauppinen et al., 2008[236]; Marreiro et al., 2017[291]).

At concentrations as high as 250-300 μM, zinc plays a crucial role in modulating the activity of glutamate NMDA receptors (Joe et al., 2018[220]). Furthermore, interactions of zinc with the gamma-aminobutyric acid (GABA) receptor, glutamatergic α-amino-3-hydroxy-5-methyl-4-isoxazole propionic acid (AMPAR) receptor, a subtype of serotonin 5-HT1A receptor, G-protein coupled 39 (GPR39) receptor, and other receptors are believed to contribute to its antidepressant properties (Petrilli et al., 2017[343]).

A meta-analysis involving ten studies encompassing 658 patients diagnosed with schizophrenia and 1,008 control subjects revealed that serum zinc levels were significantly lower in individuals with schizophrenia than in control individuals (Joe et al., 2018[220]). Notably, the decrease in zinc levels was particularly evident among newly diagnosed and drug-naïve patients.

Zinc serves as a crucial element in many enzymatic reactions and plays a significant role in systemic physiology, particularly in mediating antioxidant and anti-inflammatory responses (Mocchegiani et al., 2005[311]). Both in vivo and in vitro cell culture studies have demonstrated that zinc deficiency is correlated with increased levels of oxidative stress markers, such as thiobarbituric acid-reactive substances (TBARS) and protein carbonyls (PCs), as well as increased levels of inflammatory cytokines, including IL-1β, IL-6, and TNF-α (Doboszewska et al., 2016[121]).

The connection between redox, non-redox, and essential metals and the pathophysiology of psychosis, particularly in the case of schizophrenia, is still not well understood, largely because antipsychotic medications can influence the levels of a variety of metals. Recent human studies have further elucidated the connection between the levels of metals and the pathophysiology of schizophrenia. It has been reported that decreased zinc levels may contribute to the onset of psychosis by disrupting glutamate signaling, exacerbating ROS-mediated oxidative stress, and promoting inflammation (Murray et al., 2021[316]; Tabata et al., 2022[407]).

Several meta-analyses have indicated that individuals with depression exhibit reduced levels of zinc, as do those diagnosed with neurodevelopmental disorders such as autism spectrum disorder and attention-deficit hyperactivity disorder (Tabata et al., 2022[407]).

### Zinc and epigenetic alterations 

While increased levels of redox-active metals are frequently responsible for epigenetic dysregulation, in the case of non-redox zinc, paradoxically, epigenetic alterations are commonly associated with its deficiency. Zinc is a key metal cofactor for enzymes across all categories. This group encompasses epigenetically active enzymes, including class I, II, and IV histone deacetylases (HDACs) and acetylases (HATs), as well as DNA methyltransferases (DNMTs), histone methylases, and methyl-binding proteins (Cassandri et al., 2017[70]).

Homocysteine is a sulfhydryl-containing amino acid intermediate important in the biosynthesis of methionine and cysteine. Homocysteine is converted to methionine, which is subsequently S-adenosylated to produce S-adenosylmethionine (SAM) and acts as the primary methyl donor for various methylation reactions occurring within cells. These reactions are regulated by two essential enzymes, methionine synthase (MTR) and betaine homocysteine methyltransferase (BHMT), both of which are zinc-dependent methyltransferases. BHMT is a cytosolic enzyme that relies on zinc and facilitates the transformation of betaine to dimethylglycine and homocysteine to methionine. Zinc deficiency can impair the functionality of these enzymes, leading to reduced DNA methylation levels.

Zinc deficiency has been linked to DNA hypermethylation (Davis and Uthus 2004[108]). This phenomenon can be explained by the impact of zinc on the activity of methionine synthase, an enzyme that facilitates the conversion of homocysteine into methionine, thereby supplying essential substrates for DNA and histone methylation.

One of the factors responsible for the disrupted regulatory pathways in bone development is zinc deficiency, which is associated with epigenetic factors. The link between zinc and the epigenome is mediated by the multizinc-finger enzyme Trps1 (Wessels et al., 2017[451]). The Trps1 protein is a regulator of histone deacetylation and interacts with two histone deacetylases, Hdac1 and Hdac4, thereby increasing their activity. This process is dependent on zinc levels. Dysfunction of TRPS1 leads to the accumulation of prometaphase cells due to impaired binding of HP1 and chromatin condensation in chondrocytes lacking Trps1. Additionally, Trps1 may play a role in the regulation of mitosis in T cells. Mutations in the TRPS1 gene cause “tricho-rhino-phalangeal syndrome” (TRPS), an autosomal dominant skeletal dysplasia.

Physiological zinc levels play a crucial role in activating immune cells. Zinc deficiency can exacerbate inflammatory responses and subsequently modify the DNA methylation patterns of genes critical for the release of proinflammatory cytokines (Wong et al., 2015[454]; Gu and Zhang, 2019[170]).

The maternal epigenome plays a crucial role in optimal embryonic development (Corry et al., 2009). The epigenome of the oocyte undergoes significant remodeling throughout oogenesis. In this context, chromatin methylation is an important step in epigenetic programming during this process. Zinc deficiency during pregnancy is known to negatively affect fetal development.

Animal models revealed that a zinc-deficient diet several days before ovulation significantly disrupted oocyte chromatin methylation and preimplantation development (Tian and Diaz 2013[420]). Notably, histone H3K4 trimethylation and global DNA methylation levels in oocytes due to zinc deficiency were markedly reduced. The supplementation of animals with a common co-substrate involved in methyl group transfer, S-adenosyl methionine (SAM), restored H3K4 methylation. These findings indicate that zinc deficiency results in diminished oocyte chromatin methylation, highlighting the importance of zinc in reproductive health.

Zinc finger proteins are a highly abundant and diverse group of proteins encoded by ~ 5% of the human genome (Kamaliyan and Clarke 2024[232]). Both the functionality and expression of the zinc-finger proteins ZNF804A and ZNF326 are affected by zinc levels through epigenetic pathways (Girgenti et al., 2012[160]). A single-nucleotide polymorphism in ZNF804A has been associated with combined bipolar disorder and schizophrenia. Immunochemical analysis revealed that ZNF804a influences the transcriptional activity of four genes associated with schizophrenia and interacts with chromatin near the promoters of two schizophrenia-related genes. These results indicate that ZNF804a could play a role in regulating a transcriptional network linked to schizophrenia-associated genes.

## Conclusions

This review discusses the role of redox-active iron, copper, manganese, and redox-inactive zinc in maintaining cellular physiological functions and homeostasis. Dysregulated redox-metal homeostasis results in the metal-driven formation of ROS via the Fenton reaction, interaction with sulfhydryl protein groups, lipid peroxidation, DNA damage, and protein oxidation. Common mechanisms involving Fenton generation appear to involve redox-active iron, copper, and manganese (Me = metal) and can be described by a set of equations


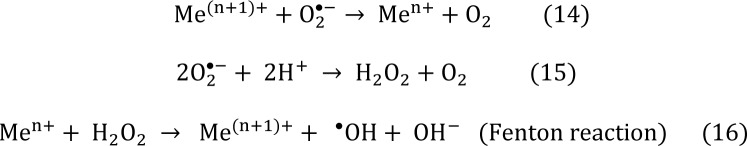


Dyshomeostasis of redox metal ions is associated with the pathogenesis of cancers; lung, renal, and gastrointestinal diseases; neurodegenerative disorders (Alzheimer's disease, Parkinson's disease, and Huntington's disease); mental disorders (schizophrenia); and a variety of cancers.

Cancer and neurodegenerative disorders, such as Alzheimer's disease, are characterized by several distinct pathologies; however, oxidative stress is a unifying factor. The increased ROS production in cancer cells results from oncogenic signaling and/or metabolic irregularities.

Hydroxyl radicals (^•^OH) are key species responsible for the oxidation of DNA, and various oxidized nucleosides have been identified. Among the nucleobases, guanine is particularly susceptible to oxidation by ROS. One of the best-quantified forms of DNA modification is 8-hydroxy-2′-deoxyguanosine (8-OHdG), which arises from the oxidative damage caused by 2′-deoxyguanosine and can lead to a G:C-T:A mispairing mutation, which is associated with the development and progression of tumors, cellular aging, and degenerative diseases. Elevated levels of 8-OHdG have been observed in various cancer tissues of ovarian, breast, prostate, nonsmall cell lung, esophageal, and colorectal cancers, whereas the surrounding healthy tissue contains low or negligible levels of this adduct.

Direct evidence indicating increased levels of redox metal-induced oxidative stress in Alzheimer's disease is substantiated by several key observations: (i) dysregulated homeostasis of redox-active iron and copper and nonredox zinc; (ii) redox metal-catalyzed formation of reactive oxygen species (ROS); (iii) elevated levels of lipid peroxidation products such as 4-hydroxynonenal, a byproduct of lipid peroxidation found in the ventricular fluid of AD patients; (iv) increased levels of oxidized proteins and DNA; and (iv) impaired energy metabolism in the Alzheimer's brain.

Maintaining redox homeostasis is essential for regulating numerous cellular signaling pathways, including the proper functioning of mitochondria and the endoplasmic reticulum, calcium homeostasis, autophagy, protein folding, and overall proteostasis. Redox-sensitive signaling pathways, such as the NF-κB, MAPK, and Nrf2 pathways, form an intricate network that governs cellular responses to redox metal-induced oxidative stress and inflammation. The Nrf2 pathway is primarily responsible for mediating antioxidant defenses, whereas the NF-κB and MAPK pathways play roles in both proinflammatory and stress-related responses.

Disruption of redox metal homeostasis may negatively affect the cellular redox balance and redox-sensitive proteins and interfere with redox-related processes, ultimately resulting in the development of a variety of oxidative stress-related diseases.

Dysregulation of non-redox zinc homeostasis may play a significant role in neuronal damage and neuronal apoptosis, potentially resulting in impairments in learning and memory. Additionally, disrupted zinc homeostasis is proposed to be a contributing factor to neurodegenerative disorders such as Alzheimer's disease, Parkinson's disease, Huntington's disease, and various other neurodegenerative conditions. The capacity of zinc as an antioxidant to mitigate oxidative stress may stem from two primary mechanisms: (i) protecting the sulfhydryl groups in proteins and enzymes from oxidative damage or (ii) suppressing the production of hydroxyl radicals from hydrogen peroxide by inhibiting the formation of ROS, which involves the antagonism of redox-active transition metals. Additionally, zinc plays a role in interactions with various elements of the immune system.

## Declaration

### Authorship contribution statement

Klaudia Jomova: Writing - review & editing, Writing - original draft, Visualization, Conceptualization. Suliman Y. Alomar: Writing - review & editing, Visualization. Richard Valko: Visualization, Writing - review & editing. Eugenie Nepovimova: Writing - review & editing, supervision, conceptualization. Kamil Kuca: Writing - review & editing, visualization, project administration, conceptualization. Marian Valko: Writing - review & editing, visualization, project administration, conceptualization.

### Conflict of interest

None.

### Funding

The authors thank the Deanship of Scientific Research, King Saud University, for funding through the Vice Deanship of Scientific Research Chairs and the Research Chair of Doping. This work was supported by projects Excelence PrF 2208/2024-2025, Excelence FIM 2203, MH CZ - DRO (UHHK, 00179906) and CZ.10.03.01/00/22_003/0000048. The authors also thank the Scientific Grant Agency of the Ministry of Education, Science and Sport for funding (grants VEGA 1/0542/24 and VEGA 1/0418/24).

### Using Artificial Intelligence (AI) 

We declare that no artificial intelligence (AI)-assisted technologies (e.g., Large Language Models [LLMs], chatbots, or image creations) were used in the submission process to complete the manuscript.
